# Unraveling the concepts of distress, burnout, and depression in type 1 diabetes: A scoping review

**DOI:** 10.1016/j.eclinm.2021.101118

**Published:** 2021-08-28

**Authors:** Dona A. Kiriella, Sumaiya Islam, Olutobi Oridota, Nancy Sohler, Coralie Dessenne, Carine de Beaufort, Guy Fagherazzi, Gloria A. Aguayo

**Affiliations:** a Community Health and Social Medicine Department, CUNY School of Medicine, New York, NY, United States; b Department of Population Health, Luxembourg Institute of Health, Strassen, Luxembourg; c Department of Paediatric Diabetes and Endocrinology, Paediatric Clinic, Hospital Centre of Luxembourg, Luxembourg, Luxembourg; d Department of Paediatric Endocrinology. Free University Brussels, UZ-VUB, Brussels, Belgium; e Deep Digital Phenotyping Research Unit, Department of Population Health, Luxembourg Institute of Health, Strassen, Luxembourg

**Keywords:** Type 1 diabetes, Depression, Diabetes distress, Fear of hypoglicaemia, Diabetes burnout, Exhaustion, Psychological burden, Diagnostic questionnaires, Selfmanagement, Insulin pump

## Abstract

**Background:**

Psychological complications are frequent in type 1 diabetes (T1D) but they might be difficult to distinguish one from the other in clinical practice. Our objective was to study the distinguishing characteristics, overlaps and their use in the literature between three concepts of T1D: depression, diabetes distress (DD) and diabetes burnout (DB).

**Methods:**

A scoping review (PRISMA guidelines) performed in three databases (PubMed/MEDLINE, PsycInfo, Web of Science) with the keywords: T1D, depression, diabetes and burnout, from January 1990 to June 2021. We selected original studies with participants with T1D, which reported depression, DD, or DB. We extracted information about the concepts, their sub-concepts and screening tools.

**Findings:**

Of the 4763 studies identified, 201 studies were included in the study. Seventy-three percent, 57% and 45% of sub-concepts do not overlap in depression, DD, and DB, respectively. We observed overlap between depression (27%)/DD (27%) and between DD (20%)/DB (50%).

**Interpretation:**

A number of sub-concepts distinguish depression and DD. Overlaps between concepts suggest that a more precise definition is still lacking. DB is still a relatively new concept and more research is needed to better understand how it can present itself differently, in order to personalize care in comparison to those having DD.

**Funding:**

This project was supported by the MSDAvenir Foundation (Project World Diabetes Distress Study).

**Research in context**

**Evidence before this study**

Psychosocial issues are among the most common complications associated with type 1 diabetes and have an impact on it. Depression and diabetes-related distress are frequently described and associated with poorer metabolic outcomes. Diabetes burnout has recently emerged in the literature as relevant for people with type 1 diabetes. From the literature it does not seem clear whether these concepts are entirely separate entities or if they overlap. Before starting this study, we performed a rapid review in PubMed and found no scoping reviews or systematic reviews that analyze the concepts of depressive distress and burnout in type 1 diabetes.

**Added value of this study**

This scoping review is the first and most comprehensive study synthesizing literature related to the use of three concepts in studies mentioning depression, diabetes distress and diabetes burnout among people living with type 1 diabetes. This includes how the concept was defined in each study, the description of unique and overlapping sub concepts for each concept and the range of scales and other methods of assessment.

**Implications of all the available evidence**

We have found that while there are overlapping sub-concepts of depression and diabetes-related distress, these psychological issues represent different concepts. It is not clear whether diabetes burnout represents a new psychosocial problem associated with diabetes or is part of the concept of diabetes distress. Clinicians who observe patients with type 1 diabetes should assess psychological issues with adequate tools for each condition to best address their psychosocial condition due to type 1 diabetes.

Alt-text: Unlabelled box

## 1 Introduction

People living with T1D are challenged with handling a chronic condition that demands structure, self-discipline and repeated health care contacts [1]. Due to the possible complications and complex management of T1D (which involves daily management of insulin adjustment to food intake and physical activity, carb counting, preventing and managing hypoglycaemia, and dealing with the health system), people living with T1D are more at risk of psychological problems than their healthy peers [2]. Psychological comorbidity can have a significant impact on blood sugar control, motivation, and quality of life [3,4]. Accordingly, it is important to diagnose, assess, and treat psychological disorders in T1D [4]. While self-care behavior is essential for people living with T1D, it is overshadowed by the development of psychiatric morbidity [5]. Psychosocial conditions that arise after diagnosis of T1D are associated with poor glycaemic control [6] and an increased demand for psychological interventions for people living with T1D [7].

The impact of diabetes on psychosocial aspects is paramount. Depression, diabetes distress and diabetes burnout are three concepts that are often referred to and used interchangeably when quantifying the psychosocial impact of T1D [8]. Depression refers to feelings of sorrow, hopelessness, worthlessness, fatigue and difficulty concentrating [9,10]. The prevalence rate of depression is three times higher in people living with T1D; depression inhibits appropriate potential diabetes management and glycaemic control [6,11].

Diabetes distress, which, unlike depression, is not a psychiatric disorder, refers to the negative emotions experienced by people living with diabetes [12]. It includes emotional stress, emotional and behavioral challenges generated by the burden of disease self-management, and ongoing worries that are associated with T1D [9,13,14]. About 40% of persons with T1D report diabetes distress [13,15].

Finally, diabetes burnout has been associated with barriers to adherence to diabetes treatment and in consequence to glycaemic control [16]. Diabetes burnout is characterized by feelings of mental, emotional, as well as physical exhaustion of being affected with diabetes [17]. Studies show that patients often not only feel drained and hopeless, but also have difficulty with self and diabetes care, and experience detachment from self and support systems, leading to an increasing feeling of powerlessness [18]. One study that assessed barriers to medication use in type 2 diabetes (T2D) reported that 36% of study participants identified the feeling of having diabetes burnout [19].

While these three concepts are similar and often used interchangeably, it is not clear that they are overlapping. For example, a recent systematic review investigated associations of concepts of depression and burnout in general (not focused on diabetes) and found positive associations but no clear overlaps [20]. However, this is not been specifically applied to T1D.

Using a systematic scoping review with a reproducible methodology, we aimed to give a very broad overview of these three psychosocial concepts in the T1D medical literature to understand how these concepts have been defined or operationalized. We aimed to disentangle these concepts and to explore their assessment tools in order to find unique characteristics or, on the contrary, overlaps.

## 2 Methods

We performed a review based on the methodology described in the Preferred Reporting Items for Systematic Reviews and Meta-Analyses (PRISMA-ScR) scoping review extension guidelines [21].

### 2.1 Search strategy and information sources

We used PubMed/MEDLINE, PsycInfo and Web of Science (WOS) databases to identify studies that define psychological issues that are prevalent in people living with T1D and their health outcomes. A trained research librarian developed the database searches strategies. The search was limited to studies published in English from January 1990 to June 2021. Studies with its citations that have met the inclusion criteria were imported to CADIMA, a review management software for screening [22]. Supplementary Table 1 shows the search strategy.

### 2.2 Eligibility criteria

We based our eligibility criteria applying the methodology of the Joanna Briggs Institute consisting in Population, Concept and Context [23]. We selected studies that included people living with T1D (Population) with outcome or main determinant: depression, diabetes distress or diabetes burnout (Concept) in a clinical setting or community-dwelling (Context) (Supplementary Table 2). We included original studies with available full-text. In addition, we used references from review studies to find additional original studies.

### 2.3 Study selection

Three independent reviewers (DK, SI, and OO) screened and reviewed published literature in triplicate. The studies were screened in two stages: (1) title and abstract and (2) full text. DK, SI and OO performed initial screening with title and abstracts. We included the studies that met the initial inclusion criteria based on title and abstract for full text screening. All three independent reviewers conducted full text screening using the same inclusion criteria and then, they performed data extraction. Conflicts of the screened studies were discussed among the independent reviewers. The principal supervisor (GA) managed the disagreements after the reviewers’ discussions.

### 2.4 Data extraction

DK, SI and OO independently extracted the data from the selected articles using the Google Sheets software. We based our data extraction sheet on the Joanna Briggs Institute guidelines [23] with the following information to extract: first author, title, year of publication, country, main objective of the study, participant characteristics (sex, current age, age at diagnosis of T1D), group characteristics (diabetes duration, HbA1c, insulin pump, chronic complications), study design, population characteristic, concept, measuring scale and context. DK, SI and OO independently extracted the data from the selected studies. We extracted the main concepts categorized as depression, diabetes distress and diabetes burnout and their scales for measuring the concepts.

In addition, we extracted words and sentences describing the concepts. This was done by DK and OO and reviewed by SI using words and phrases from concept definitions in the included studies. Where there were no explicit concept definitions, we used questionnaires and/or scales assessing concepts. We extracted semantic descriptions of each question/statement from all of the questionnaires/scales. Then, the words and sentences were simplified, harmonized and shortened. We reported the frequency of words and phrases for each concept. This information was used to build the figures in this article that explain the concepts. The results of the data extraction were discussed, and inconsistencies were resolved with GA. In addition, an analysis was performed by separating the extracted semantic descriptions by questionnaires/scales and authors’ descriptions/definitions. To plot the word cloud for each concept, we used text-mining techniques consisting that the extracted text was copied as many times as it was referenced and saved to a text file and then plotted.

We also calculated the median differences in reported mean age, percentage of females, duration of diabetes, and HbA1c among studies focusing on depression, distress, and depression. We used a nonparametric Kruskall-Wallis test and considered the p values to be significant with an alpha value < 0.05.

We used RStudio version 1.3.1093 with packages “ggplot2”, "ggpubr», tidyverse”, “tm”, “SnowballC”, “wordcloud”, “RColorBrewer”, “VennDiagram” and “grid”.

### 2.5 Role of the funding source

The study sponsor played no role in the design of the study; nor in the collection, analysis and interpretation of data; nor in the drafting of the report; and neither in the decision to submit the article for publication. DK, SI, OO and GA had full access to all the data in the study and all authors accept responsibility to submit for publication.

## 3 Results

**Fig. 1:**
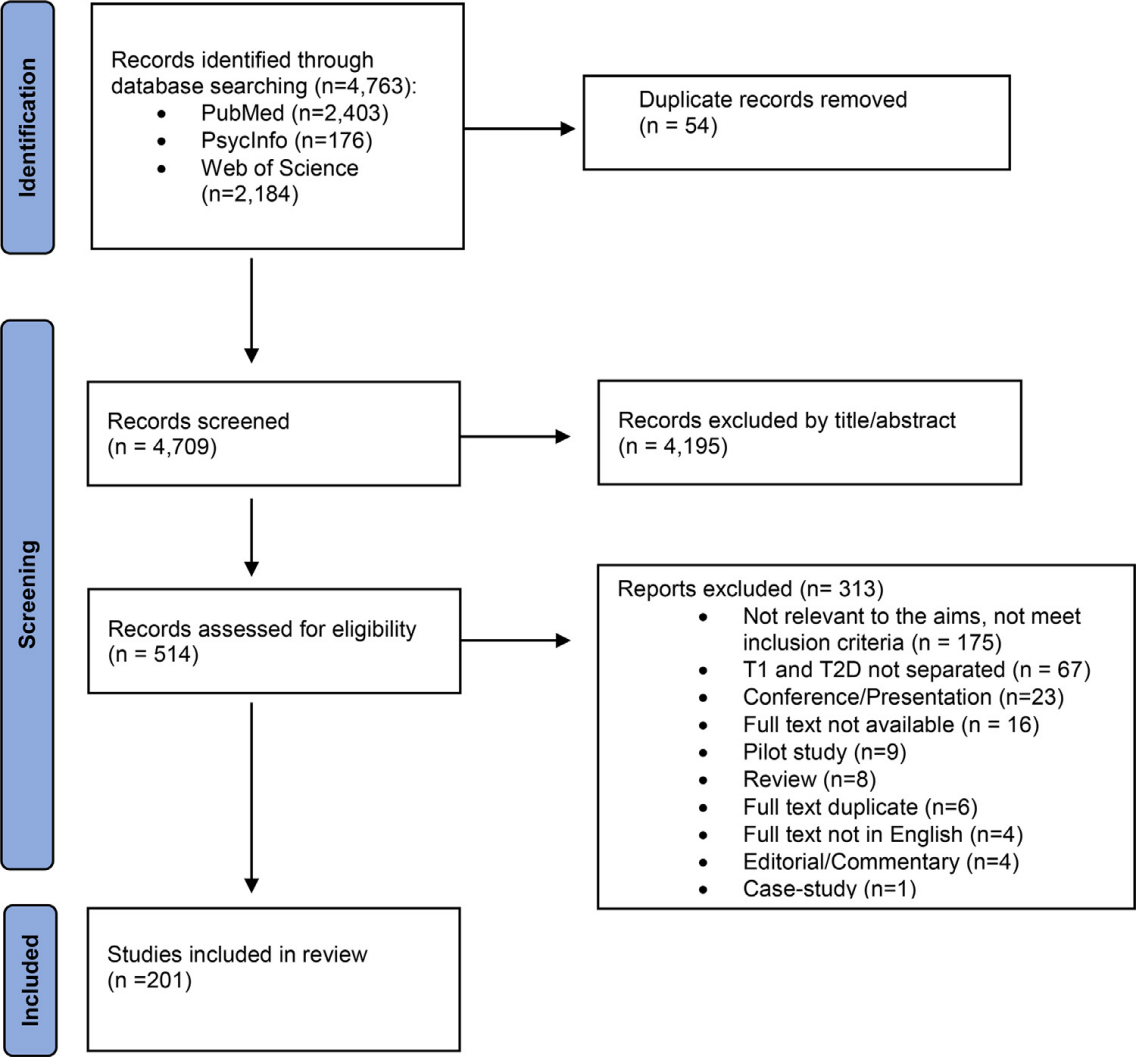
PRISMA Flowchart. Number of records identified, included and excluded with reasons.

The literature search of the electronic databases yielded 4763 publications (PubMed, *n* = 2403; PsycInfo, *n* = 176; WOS, *n* = 2184). After removing duplicates, 4709 titles and abstracts were screened for eligibility. Of the 514 studies eligible for full text screening, 313 studies were further excluded. The reasons for exclusion are shown in the PRISMA Flowchart (Fig. 1). Finally, we included 201 studies for full-text analysis.

### 3.1 Study characteristics

The total number of participants included in the studies in this review was 218,185 and ranged from 11 participants to 79,067. The average total percentage of women was 55% and was reported in 94% of the studies. One hundred seventy-two (86%) studies included only T1D and 29 (14%) studies both T1D and T2D. Mean age was 29 (SD = 15) years and was reported by 93% of studies.

**Fig. 2:**
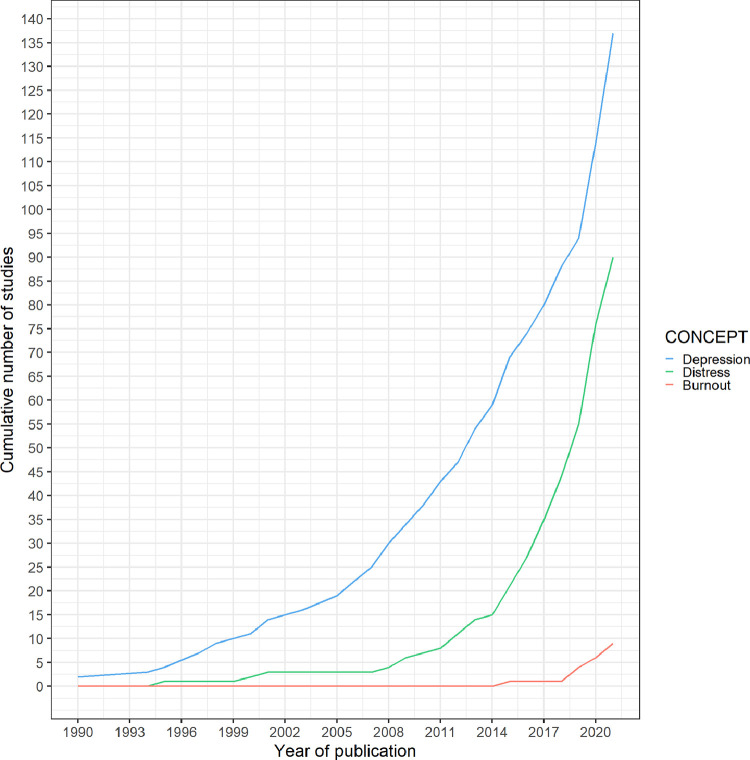
Cumulative number of studies by year of publication. The blue line shows studies in depression, the green line in diabetes distress and the salmon line in diabetes burnout.

Two studies were published as early as 1990. One hundred thirty-four studies (67%) were published in 2015 and after (Fig. 2). Most articles were published in the USA (*n* = 87, 43%), in the UK (*n* = 17, 8%), in Germany (*n* = 14, 7%), and in the Netherlands (*n* = 12, 6%) (Supplementary Fig. 1). One hundred four (52%) studies reported adult participants aged 18 years old and over. Participants in the included studies were recruited from various settings. The context of most studies (*n* = 146, 73%) were in outpatient clinics, inpatient settings were reported in (*n* = 25, 12%) studies. (Supplementary Table 3). One hundred seventy two studies (86%) were observational and (*n* = 29, 14%) were interventional. The most frequent study design was cross-sectional (*n* = 113, 56%).

### 3.2 Overview of concepts of depression, diabetes distress and diabetes burnout

When extracting the semantic descriptions of the concepts, questionnaires were used 151 times and descriptions from authors were used 94 times. In total, there were 1730 words and sentences from the articles related to the concept. Analysing the main concept by study, depression was the most mentioned concept. One hundred thirty-seven studies (68%) mention depression as one of the main outcomes and 106 studies (53%) mention depression as the outcome of the study. Ninety studies (45%) mentioned diabetes distress as one of the main outcomes and (*n* = 59, 29%) mentioned it as the main outcome. Nine studies (4%) mentioned diabetes burnout as one of the main outcomes and five studies (2%) mentioned it as the main outcome. Analysing all concepts by study, some studies focused on more than one concept, depression and diabetes distress (*n* = 27, 13%), and depression, diabetes burnout and diabetes distress, (*n* = 4, 2%) (Supplementary Table 3).

Diabetes duration was reported in 154 (77%) studies and had a median (IQR) of 12 (6, 19) years. Diabetes duration was longer in studies focused exclusively on diabetes distress (median (IQR) 17 (8, 22) years or diabetes distress and depression (median (IQR) 16 (7, 18) years) than those focused exclusively on depression (median (IQR) 7 (6, 18) years) and was reported in one study within the burnout concept (20 years) (*p*-value = 0.009516). HbA1c was reported in 171 (85%) studies and had a median (IQR) of 8.4 (7.8, 8.9)%. HbA1c had a median (IQR) of 8.7 (8.0, 9.1)%, 8.2 (7.7, 8.8)%, and 8.2 (7.8, 9.0)% in studies which focused exclusively on depression, diabetes distress and focused on depression and diabetes distress, respectively. There was only one study focused on burnout that reported HbA1 (9.3%). Studies focusing on depression, diabetes distress, diabetes burnout and both depression and diabetes distress reported mean ages of 26, 32, 32 and 30 years, respectively (*p* value = 0.08).

Hypoglycaemia was reported in (*n* = 39, 19%) studies. By main concept, hypoglycaemia was reported in (*n* = 20, 19%) of studies with the depression concept, in (*n* = 15, 25%) studies with the diabetes distress concept, in four (15%) studies with both depression and diabetes distress concepts and was not reported in diabetes burnout studies. Insulin pumps were reported in (*n* = 88, 44%) studies. It were reported in (*n* = 40, 38%) studies with the depression concept, in (*n* = 31, 53%) studies with the diabetes distress concept, in (*n* = 14, 52%) of studies with both depression and diabetes distress concepts and in two (40%) studies with the diabetes burnout concept. Chronic complications were reported in (*n* = 56, 28%) studies. They were reported in (*n* = 30, 28%) studies with the depression concept, in (*n* = 19, 32%) studies with the diabetes distress concept, in seven (26%) studies with both the depression and diabetes distress concepts and were not reported in diabetes burnout studies.

### 3.3 Depression

Among studies that examined depression (*n* = 137, 68%), various assessment tools were used (19 tools). The most used tools were the Children's Depression Inventory (CDI) [24] (*n* = 32, 23%), the Center for Epidemiological Studies Depression Scale (CES-D) [25] (*n* = 25, 18%), the Beck Depression Inventory (BDI) [26] (*n* = 23, 17%), the Patient Health Questionnaire (PHQ) [27] (*n* = 21, 15%), and the Hospital Anxiety and Depression Scale (HADS) [28] (*n* = 15, 11%). Most of the studies assessed depressive symptoms with self-reported questionnaires and only 6 (4%) applied the Diagnostic and Statistical Manual of Mental Disorders, 5th edition (DSM-5) [29] criteria to diagnose major depression (Table 1).

**Table 1:** Measurements (self-reported questionnaires and interviews) of depression, diabetes distress and diabetes burnout.

**Scales /Questionnaires /Interviews**	**Population**	**Items**	**Subscales, keywords or description**	**Number (%)**
**Depression (137 studies)**				
Children's Depression Inventory (CDI) [24]	Children	27	Anhedonia, Ineffectiveness, Interpersonal problems, Negative mood, Negative self-esteem	32 (23%)
Center for Epidemiological Studies Depression Scale (CES-D) [25]	Adults and children	20	Depressed mood, feelings of guilt and worthlessness, feelings of helplessness and hopelessness, psychomotor retardation, loss of appetite, and sleep disturbance	25 (18%)
Beck Depression Inventory (BDI) [26]	Adults and adolescents	13 or 21	Sad, discouraged, feeling a failure, dissatisfaction, guilty, punished, disappointed, worse, suicide, cry, irritated, loss of interest, difficulty to make decisions, unattractive, loss of effort, sleep, appetite, lose weight, worried, lost interest in sex	23 (17%)
Patient Health Questionnaire (PHQ)[27]	Adults	2, 8 or 9	Little interest, Feeling down, trouble sleep, tired, appetite, failure, concentration, restless, death	21 (15%)
Hospital Anxiety and Depression Scale (HADS) [28]	Adults	14	Tense, slowed down, enjoy, frightened, awful, appearance, laugh, restless, worrying, enjoyment, cheerful, panic, relaxed, enjoy things	15 (11%)
Structured Clinical Interview for the DSM [29]	Adults	8	Depressed mood, low interest, weight loss, slowing down, fatigue, feeling guilty, difficulty concentrating, suicidal thoughts	6 (4%)
World Health disorganization- Five Well-Being Index (WHO-5) [56]	Children and adults	5	Cheerful, calm, active and vigorous, fresh, interest	4 (3%)
Hopkins Symptom Checklist (SCL-90-R) [57]	Adults	90	Somatization, obsessive-compulsive disorder, interpersonal sensitivity, depression, anxiety, hostility, phobic anxiety, paranoid ideation, and psychoticism	4 (3%)
ICD codes [49]	Adults	–		3 (2%)
Global Severity Index (GSI-depression subscale) [58]	Adults	53	Somatization, obsessive-compulsive disorder, interpersonal sensitivity, depression, anxiety, hostility, phobic anxiety, paranoid ideation, and psychoticism	2 (1%)
Major Depression Inventory (MDI) [59]	Adults	12	Sad, loss of interest, lack of energy, less-self-confident, guilty, worth, restless, concentration, slow-down, sleep, increased or decreased appetite	2 (1%)
Psychological General Well-Being Index (PGWB) (depression subscale) [60]	Adults	22	Anxiety, Depression, Positive well-being, Self-control, General health, Vitality	2 (1%)
Mini International Neuropsychiatric Interview [61]	Adults	17	–	2 (1%)
Depression Self-Rating Scale of Children (DSRS) [62]	Children	18	Look forward, sleep well, crying, go out to play, running, tummy aches, energy, enjoy food, stick up, worth, good, enjoy things, talk, bad dreams, cheered up sad bored	1 (1%)
Behavior Assessment System for Children (BASC) [63]	Children	137	Evaluates behavior and emotional status.	1 (1%)
Positive and Negative Affect Schedule - Expanded Form (PANAS-X) [64]	Adults	60	Fear, hostility guilt, sadness	1 (1%)
Inventory of Depression and Anxiety Symptoms (IDAS) [65]	Adults	20	Suicidality, Lassitude, Insomnia, Appetite Loss, Appetite Gain, Ill Temper, Well-Being, Panic, Social Anxiety, and Traumatic Intrusions	1 (1%)
Mood and Feelings Questionnaire (MFQ) [66]	Children	33 or 13	Unhappy, don't enjoy, tired, restless, no good, cry, hart to concentrate, hate him-herself, bad person, nobody love.	1 (1%)
Well-Being Questionnaire (WBQ) [67]	Adults	22	Depression, anxiety, energy, positive well-being	1 (1%)
**Diabetes distress (90 studies)**				
Problem Areas in Diabetes Questionnaire (PAID) [30]	Adults, adolescents and children	20 (PAID adults), 26 (PAID-T adolescents), 11 (PAID-C children), and short version 5 (PAID-5)	Not having concrete goals, discouraged, scared, uncomfortable in social situations, deprivation, depressed, overwhelmed, worrying about hypoglycaemia, angry, concerned, worrying about the future, guilty, anxiety, not acceptation, dissatisfaction, lack of energy, alone, lack of support, coping with complications, burned-out	51 (57%)
Diabetes Distress Scale (DDS) [31]	Adults, adolescents and children	28 (DDS-T1) or 17 (DDS)	Emotional Burden, Physician Distress, Regimen Distress, Emotional Distress	36 (40%)
Observational Scale of Behavioral Distress–Revised (OSBD–R) [68]	Children	8	Crying, screaming, restraining, verbal resistance and information seeking, solicitation of emotional support, verbal pain expression and flail.	1 (1%)
Kessler Psychological Distress (K6) [69]	Adults	6	Nervous, hopeless, restless or fidgety, depressed, everything was an effort	1 (1%)
Fragebogen zu Alltagsbelastungen bei Diabetes (FBD) [70]	Adults	45	Everyday stress, emotions, cognition and behavior	1 (1%)
**Diabetes burnout (9 studies)**				
Maslach Burnout Inventory-General Survey (MBI-GS), exhaustion subscale [32]	Adults	9	Being overextended and exhausted by one's work	3 (33%)
Diabetes Burnout Scale (DBS) [33]	Adults	18 or 12	Mentally tired, drained, emotionally exhausted, minimum to survive, ignore diabetes, out of control	2 (22%)
Illness Identity Questionnaire (IIQ) [34]	Adults	25	Engulfment, rejection, acceptance, enrichment	1 (11%)
Motivation and Attitude Toward Changing Health (MATCH) [35]	Adults	9	Managing health problems, take care, putting energy, benefit, make changes, able to fit tasks	1 (11%)
Diabetes Empowerment Scale-Short Form (DES-SF) [36]	Adults	28	Managing the psychosocial aspects of diabetes, assessing dissatisfaction and readiness to change, and setting and achieving goals	1 (11%)

**Fig. 3:**
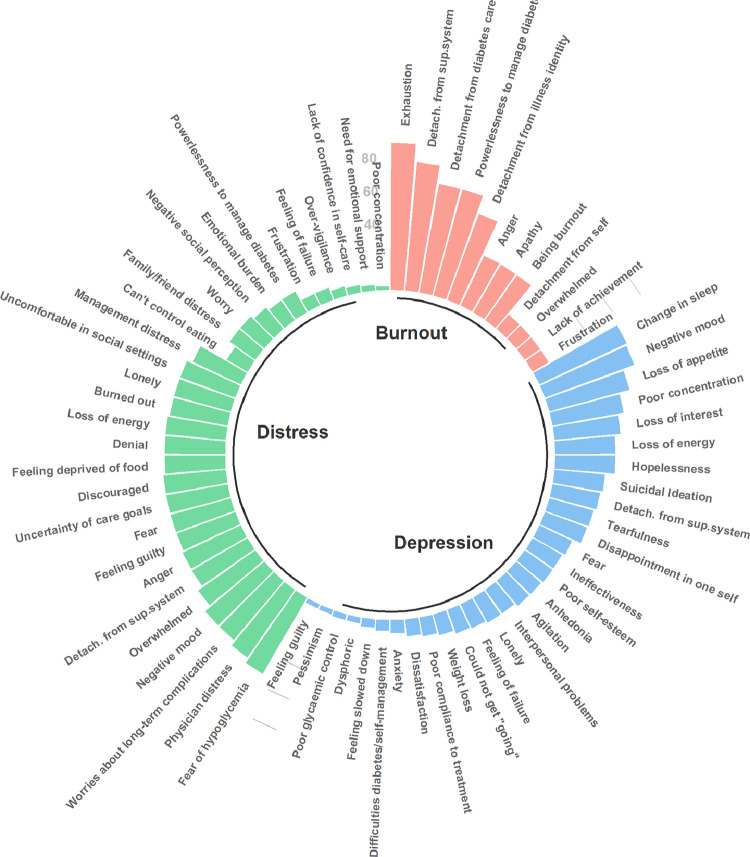
Circular bar plot of concepts of depression, diabetes distress and diabetes burnout. Bar length represents the frequency of words or phrases for each concept in percentage among the 201 included studies. Words or phrases found in studies about depression, diabetes distress and diabetes burnout are in blue, green and salmon, respectively. Abbreviation: Detach.from sup.system = Detachment from support system.

The dimensions associated with depression in T1D varied amongst the included studies. We identified 30 sub-concepts, of which (*n* = 22, 73%) were exclusive to this concept. Depression was characterized in the included articles by the following symptoms: change in sleep (*n* = 80, 58%), negative mood (*n* = 79, 58%), loss of appetite (*n* = 69, 50%), poor concentration (*n* = 60, 44%), loss of interest (*n* = 55, 40%), loss of energy (*n* = 50, 36%), hopelessness (*n* = 50, 36%), suicidal ideation (*n* = 42, 31%), detachment from support systems (*n* = 40, 29%), tearfulness (*n* = 38, 28%), disappointment in oneself (*n* = 37, 27%), fearful (*n* = 30, 22%), ineffectiveness (*n* = 28, 20%), poor self-esteem (*n* = 28, 20%), anhedonia (*n* = 27, 20%), agitation (*n* = 27, 20%), interpersonal problems (*n* = 24, 18%), lonely (*n* = 24, 18%), feeling of failure (*n* = 22, 16%), could not get "going" (*n* = 21, 15%), weight loss (*n* = 18, 13%), poor compliance to treatment (*n* = 16, 12%), and dissatisfaction (*n* = 14, 10%). Lesser concepts words for depression included anxiety (*n* = 11, 8%), difficulties with diabetes self-management (*n* = 9, 7%), feeling slowed down (*n* = 7, 5%), dysphoria (*n* = 5, 4%), poor glycaemic control (*n* = 5, 4%), pessimism (*n* = 4, 3%), and feeling guilty (*n* = 4, 3%) (Fig. 3).

### 3.4 Diabetes distress

In studies that examined diabetes distress (90 articles, 45%), various tools were used (5 tools, Table 1). Across the articles the tools that were utilized the most were the Problem Areas in Diabetes Scale (PAID) [30] (*n* = 51, 57%) and the Diabetes Distress Scale (T1- DDS and DDS) [31] (*n* = 36, 40%) (Table 1).

We identified 30 sub-concepts, of which 17 (57%) were exclusive to this concept. Diabetes distress was characterized in the included studies by the following symptoms: fear of hypoglycaemia (*n* = 48, 53%), physician distress (disappointment with physicians in charge of diabetes treatment) (*n* = 45, 50%), worries about long-term complications (*n* = 42, 47%), negative mood (*n* = 42, 47%), overwhelmed (*n* = 38, 42%), detachment from support system (*n* = 35, 39%), anger (*n* = 35, 39%), feeling of failure (*n* = 34, 38%), fear (*n* = 34, 38%), uncertainty of diabetes care goals (*n* = 34, 38%), discouraged with treatment plan (*n* = 34, 38%), feeling deprived of food (*n* = 33, 37%), denial (*n* = 33, 37%), loss of energy (*n* = 33, 37%), burned out (*n* = 32, 36%), lonely (*n* = 32, 36%), uncomfortable in social settings (*n* = 32, 36%), management distress (*n* = 29, 32%), can't control eating (*n* = 13, 14%), family/friend distress (*n* = 17, 19%), worry (*n* = 17, 19%), negative social perception (*n* = 15, 17%), emotional burden (*n* = 13, 14%), powerlessness to manage diabetes (*n* = 13, 14%), frustration (*n* = 7, 8%), feeling of failure (*n* = 7, 8%), over-vigilance (*n* = 5, 6%), lack of confidence in self-care (*n* = 4, 4%), need for emotional support (*n* = 3, 3%), and poor concentration (*n* = 2, 2%) (Fig. 3).

### 3.5 Diabetes burnout

Five scales were used to convey the facets of diabetes burnout among 9 articles, which includes Maslach Burnout Inventory-General Survey (MBI-GS) (*n* = 3, 33%) [32], Diabetes Burnout Scale (DBS) [33] (*n* = 2, 22%), Illness Identity Questionnaire (IIQ) [34] (*n* = 1, 11%), Motivation and Attitude Toward Changing Health (MATCH) [35] (*n* = 1, 11%) and Diabetes Empowerment Scale-Short Form (DES-SF) [36] (*n* = 1, 11%) (Table 1).

We identified 12 sub-concepts, of which six (50%) were exclusive to this concept. Diabetes burnout was characterized in the included studies by the following symptoms: exhaustion (ranging mentally, physically and emotionally) (*n* = 8, 89%), detachment from the support system (*n* = 7, 78%), detachment from diabetes care (*n* = 6, 67%), powerlessness as the inability for one to manage their diabetes (18) (*n* = 6, 67%), detachment from illness identity (*n* = 5, 56%), anger (*n* = 3, 33%), apathy (*n* = 3, 33%), being burnout (*n* = 3, 33%), detachment from self (*n* = 1, 11%), overwhelmed (*n* = 1, 11%), lack of achievement (*n* = 1, 11%), and, frustration (*n* = 1, 11%).

Supplementary Figs. 2 and 3 show the analysis of words and phrases by author descriptions and questionnaire items, respectively. Among words and sentences mentioned by author's descriptions, we did not find the following terms in the questionnaire items: “glycaemic control” (depression), “worry”, “emotional burden”, “frustration” (diabetes distress). Among words and sentences found in questionnaire items, we did not find the following terms in the author's descriptions: “tearfulness”, “disappointment in one self”, “interpersonal problems”, “feeling of failure”, “could not get "going", “dissatisfaction”, “dysphoric” (depression), “uncertainty of care goals”, “feeling deprived of food”, “burned out”, “uncomfortable in social settings”, “can't control eating”, “physician distress” (diabetes distress). For diabetes burnout, all words and sentences of questionnaire items were also mentioned in authors’ descriptions.

Supplementary Fig. 4 shows a word cloud of the three concepts. The most common sub-concepts in the studies are in the center of the word cloud and are represented with larger font sizes. The top ten words for depression were: “feelings”, “negative”, “diabetes”, “poor”, “lack”,” energy”, “loss”, “interest”, “mood” and “bad”. The top ten words for diabetes distress were: “diabetes”, “negative”, “emotional”, “regimen”, “management”, “distress”, “feeling”, “worries”, “burden” and “overwhelmed''. The top ten words for diabetes burnout were “diabetes”, “detachment”, “care”, “self”, “support”, “self-care”, “physically”, “tired”, “got” and, “still”.

### 3.6 Overlapping concepts

There were overlaps between the concepts of depression, diabetes distress, and diabetes burnout. Depression and diabetes distress had seven overlapping sub concepts: negative mood (60% and 48% of depression and distress studies, respectively), fear (23% and 39% of depression and distress studies, respectively), feeling of failure (17% and 9% of depression and distress studies, respectively), lonely (18% and 37% of depression and distress studies, respectively), loss of energy (38% for both depression and distress studies), feeling guilty (3% and 39% of depression and distress studies, respectively), and poor concentration (45% and 2% of depression and distress studies, respectively).

Diabetes distress and diabetes burnout had five overlapping sub concepts: powerlessness to manage diabetes (15% and 67% of diabetes distress and diabetes burnout studies, respectively), anger (40% and 33% of diabetes distress and diabetes burnout studies, respectively), burned-out (37% and 33% of diabetes distress and diabetes burnout studies, respectively), overwhelmed (44% and 11% of diabetes distress and diabetes burnout studies, respectively) and frustration (8% and 11% of diabetes distress and diabetes burnout studies, respectively).

Detachment from support systems was noted in all three concepts of depression, diabetes distress and diabetes burnout (30%, 40% and 78% of depression, diabetes distress and diabetes burnout studies, respectively).

Supplementary Table 4 shows sub concepts for each of the three concepts with and without overlapping.

## 4 Discussion

Focusing on people living with T1D, this scoping review evaluates the features and measures of the concepts of depression, diabetes distress and diabetes burnout. This study characterizes the psychosocial impacts of T1D in terms of the defined outcomes. “Change in sleep” was the most common dimension for depression, followed by “fear of hypoglycaemia” for diabetes distress and “exhaustion” for diabetes burnout. Depression and diabetes burnout hardly overlapped. Depression and diabetes distress had overlapping sub-concepts as well as diabetes distress and diabetes burnout.

We found that depression was the most studied concept and the first to be included in T1D research. It was characterized primarily by symptoms that were not specific for T1D. The current review shows that more than 50% of the studies assessing depression described this concept using “negative mood” and “loss of sleep”. According to the DSM-5, an individual must have met five of the nine criteria during a 2-week period to be classified as having major depression [29]. The descriptions of depression such as “negative mood”, “poor concentration”, “loss of appetite”, and “loss of energy” are similar to the nine criteria according to DSM-5 which include feeling sad for most of the day, diminished pleasure, weight loss or weight gain, insomnia or hypersomnia, psychomotor agitation, loss of energy, feeling of worthlessness, diminished ability to think or concentrate, and suicidal ideation.

Some symptoms of depression may overlap with symptoms of poor metabolic control such as sleep disturbances. For example, a short duration of sleep or sleep fragmentation increases the risk of developing diabetes and poor metabolic regulation in T1D, sometimes preceded by depression and can also be a symptom of hyper or hypoglycemia [37]. It is important to stress that the association between depression and poor blood sugar control may not be equal for all symptoms of depression [38].

**Fig. 4:**
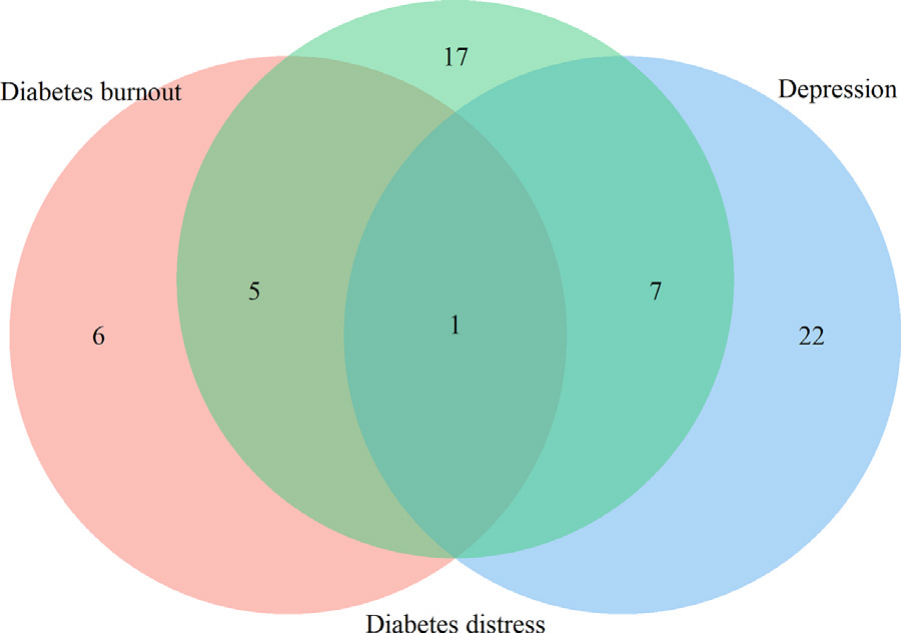
Areas of overlap among the three concepts One sentence is common for the three concepts “Detachment from support systems”. Overlapping between depression (blue) and diabetes distress (green) is in seven more sub concepts: “Fear”, “Feeling guilty”, “Feeling of failure”, “Lonely”, “Loss of energy”, “Negative mood” and “Poor concentration”. Overlapping between diabetes distress (green) and diabetes burnout (salmon) is in five more sentences/words: “Anger”, “Burned-out”, “Frustration”, “Overwhelmed” and “Powerlessness to manage diabetes”. Twenty-two (73%), 17 (57%) and five sentences (45%) do not overlap in depression, diabetes distress and diabetes burnout, respectively.

Diabetes distress and diabetes burnout were characterized by more specific symptoms related to T1D. Fear of hypoglycaemia was most commonly seen in relation with diabetes distress. Studies have shown that management of T1D at optimal glycaemic levels can cause distress with constant blood glucose monitoring and concern over coping an acute hypoglycaemic event [39]. Positive associations between fear of hypoglycaemia and HbA1c have been demonstrated among those treated with insulin injections [40]. Conceptual words such as “management distress”, “fear”, and “worry”, can be used to describe the anxiety and stress that people living with T1D might experience after their diabetes diagnosis (Figs. 3 and 4). These conceptual words are in line with the definition that Fisher gives for diabetes distress [14]. Both diabetes distress and depression were described as negative mood, fear, guilt, and loneliness. We found that for depression the central sub-concepts were unrelated to diabetes, unlike diabetes distress and diabetes burnout where “diabetes” is the most common word. Fisher et al. proposes emotional distress as an ongoing central dimension linking diabetes distress and depression [8].

Indeed, depression in T1D has been reported to be over diagnosed using the patient health questionnaire with false positives, which can be interpreted as being due to a high prevalence of diabetes distress [41]. The results of our study suggest that although there are overlaps between depression and diabetes distress, these appear to be different entities. This is in agreement with Snoek et al. who found that although the two concepts are related to poor glycaemic control, they are conceptually different, with depression being defined by symptoms and distress as an ongoing response to difficulties in managing diabetes [38]. However, in the case of diabetes distress and diabetes burnout both concepts centered on diabetes as sub concept, doubts remain as to whether these concepts are interchangeable or whether one is an aggravation of the other.

In addition, we found that diabetes duration was higher in the studies about diabetes distress. This is in agreement with a recent study that found an association of diabetes distress with a longer diabetes duration, younger age, use of insulin and T1D [42].

We found that PAID scale was the most commonly used scale among studies examining diabetes distress (57%), followed by DDS (40%). These are precisely the scales identified by the review by Dennick et al. that best operationalized diabetes distress among 53 diabetes-specific measures [12]. PAID focuses on distress concerns arising from eating, existing complications and hypoglycaemia, while DDS focuses on physician-related distress and difficulties in diabetes management [43]. The newly constructed T1-DDS demonstrates seven sources of distress including: powerlessness, negative social perceptions, physician distress, friend/family distress, hypoglycaemia distress, management distress, and eating distress; these facets of distress specific to T1D are comparable to the definitions and concepts identified by the included studies. [14].

Although diabetes burnout was previously described in the 1980s [44], we found very few studies that fully met the inclusion criteria for this study. Most of these studies were published by the same research group [17,18,33,[45], [46], [47], [48]]. Diabetes burnout is often used synonymously with diabetes distress, but they are said to be two separate concepts, described by Abdoli et al. as an inappropriate response to diabetes distress [17]. We see this in our study where 21 studies assessing diabetes distress describe this concept as “being burned out” (Fig. 3). Exhaustion was the defining element of diabetes burnout among the included studies. Studies distinguished burnout from diabetes distress by covering a wider range of elements including powerlessness and hypoglycaemia worry [14,17].

Burnout is currently not considered part of the DSM-5, while the International Classification of Diseases [49] recognizes burnout as a factor influencing the health status of those who are employed or unemployed with three dimensions including feelings of exhaustion [50]. Out of the five scales for diabetes burnout within the current review, the Illness Identity Questionnaire (IIQ) scale depicts the demands of T1D for constant optimal glycaemic levels as it becomes integrated into daily life and soon as part of their identity [34]. The use of the Maslach Burnout Inventory (MBI-GS) was originally intended to observe work burnout in employees who are under chronic stress; hence, components of the MBI-GS can be reflected into people living with T1D where chronic stress can be emotional draining leading to emotional exhaustion, depersonalization, and reduced personal accomplishment [51]. When comparing to the studies assessing depression and diabetes distress, few studies have addressed diabetes burnout. The results from our study highlight the need for more research on diabetes burnout to distinguish from diabetes distress. Finally, based on qualitative studies and on expert opinion, a recent publication proposed a specific scale for diabetes burnout. This scale shows high correlations with depression and diabetes distress and is a good predictor of HbA1c levels [33].

The results demonstrated that detachment from support systems overlaps depression, diabetes distress and diabetes burnout. Type 1 diabetes comes with the burden of extensive monitoring and insulin administration, and in turn, people living with T1D may feel less support from their families due to breaks in activity for insulin injections or glucose monitoring [52].

In a review by Snoek et al. in 2015, [38] it was found that depression and diabetes distress instruments were strongly correlated, from *r* = 0.63 (CES-D and PAID) [53] to *r* = 0.04 (MINI and DDS) [54]. However, Snoek pointed out that much of the variance in diabetes-related distress remained unexplained, suggesting that these two concepts were distinct. The results of this scoping review using a structured and strict methodology are consistent with these results showing that despite the overlap of concepts, depression and diabetes-related distress appear to be different constructs and may not be interchangeable.

It is important to emphasize that depression is a psychiatric condition; therefore, it is naturally classified as the presence or absence of depression. Its definition is based on symptoms and not on etiology. The fact that depression was assessed using self-reported questionnaires, which include symptoms described in the DSM-5 and symptoms of psychological distress, suggests that what it is measured is depressive symptoms mixed with symptoms of distress, not depression as such [8]. It is different from diabetes distress, which is an adaptive emotional response to disease burden with an etiology-based (diabetes-related) concept. In addition, depression is very heterogeneous in many aspects: phenotype, extent, symptoms, course over time, etiology and others) [55]. Regarding sources of heterogeneity, we found 19 different instruments claiming to measure depression and only four and five for diabetes distress and diabetes burnout, respectively.

In depression and diabetes distress, we found that most of the words and phrases found in the author's descriptions were also found in questionnaires. However, some words and phrases that were found in the questionnaires were not mentioned in the author's descriptions. For diabetes burnout, all of the words and phrases were the same in both sources. This result suggests that the concepts of depression and diabetes-related distress are more fully and precisely defined by questionnaires. The fact that diabetes burnout has been defined uniformly by both sources is likely due to the fact that there are not enough questionnaires for this concept and most of the evidence comes from the same research group.

This review strictly followed PRISMA's methodology for scoping reviews (Supplementary Table 4) with three database searches. It gives a very comprehensive and broad view of the literature on three important concepts in diabetes care. However, this study has some limitations. For example, when screening the literature, we included a few articles examining both patients with T1D as well as T2D. Articles that included both T1D and T2D were screened to ensure that the T1D cohort consisted of more than 50% of the population. Therefore, if the study population was mostly T2D, the study was not included. Another limitation of the current review is that it is solely limited to the three psychosocial concepts of diabetes - depression, diabetes distress, and diabetes burnout. Examining other psychosocial outcomes that are concurrent with T1D may elucidate certain links between the concepts and yield further implications for patients afflicted with T1D. Additionally, a limitation of this study is that the included studies showed great heterogeneity in how they measured study concept, design, and quality. In addition, we included only studies in English and this could limit the generalization of our results. Finally, we analysed the words and concepts taking information not only from the validated questionnaires but also from the descriptions of the authors. This may increase the heterogeneity of the results, but at the same time does not exclude other sources of information such as authors’ description based on qualitative research.

The results of this study help clarify the underlying concepts of three psychological problems common in T1D, paving the way for a distinction between depression, diabetes distress, and diabetes burnout. Depression is a very common psychological comorbidity in T1D, although it is nonspecific. This condition is assessed with a plethora of questionnaires, which increases the heterogeneity of the concept and increases the likelihood of overlap. Diabetes distress is also common but specific, and despite some overlap with depression, it seems an independent and different concept. As diabetes burnout is emerging as a concept in T1D, is frequently mentioned in studies of diabetes distress and presents overlapping sub-concepts, there is a need to better conceptualize it to differentiate it from diabetes distress. When depressive symptoms are detected with a self-reported instrument, clinicians should be aware that due to the possible overlap with diabetes distress, this diagnosis should also be evaluated. The same situation can occur when diabetes distress is detected, and in addition, in this case, a diagnosis of diabetes burnout should also be screened. An early differentiation of these concepts will best help to recognize and treat the psychosocial problem with the right approach. Next steps are to perform data-driven analyses of qualitative studies and expert opinion with the objective of further refine these concepts. Another step should be to further analyze diabetes burnout and its differentiation from diabetes distress in clinical studies.

## Funding

This project was supported by the MSDAvenir Foundation (Project World Diabetes Distress Study).

## Contributors

GF planned the search. CD did the research strategy. DK, SI and OO performed the literature search, extracted and summarized the data. GA resolved the conflict in the selection and extraction process, verified the extraction and made the figures. DK and GA have made tables. GA, GF, CDB and GF edited the revised manuscript.

## Data sharing

Data collected for this scoping review have already been published in other studies. Data extracted from these published articles will be made available to others upon request. There is no individual participant data due to the nature of this scoping-review. The data will be made available beginning 3 months and ending 5 years following article publication. The data will be made available to researchers who provide a methodologically sound proposal. Proposals should be directed to gloria.aguayo@lih.lu; to gain access, data requestors will need to sign a data access agreement.

## Declaration of Competing Interest

The authors declare no conflict of interest.

## Appendix Supplementary materials

Image, application 1

Image, application 2
