# Supplementary material for: Unraveling the concepts of distress, burnout, and depression in type 1 diabetes: A scoping review
Source: eClinicalMedicine. 2021 Aug 28;40:101118. doi: 10.1016/j.eclinm.2021.101118 (PMC8408521; doi:10.1016/j.eclinm.2021.101118)
Supplement: Supplementary file 1 [file mmc1.docx]

Supplementary material

**Contents**

[Supplementary Table 1. Search strategy 2](#_Toc79753137)

[Supplementary Table 2. Inclusion criteria 4](#_Toc79753138)

[Supplementary Table 3. Summary of all studies 5](#_Toc79753139)

[Supplementary Table 4. Frequency and overlapping of sub concepts: depression, diabetes distress and diabetes burnout 18](#_Toc79753140)

[Supplementary Figure 1. Number of publications by regions of the world 20](#_Toc79753141)

[Supplementary Figure 2. Circular bar plot of concepts of depression, diabetes distress and diabetes burnout by authors’ descriptions 21](#_Toc79753142)

[Supplementary Figure 3. Circular bar plot of concepts of depression, diabetes distress and diabetes burnout by questionnaire items 22](#_Toc79753143)

[Supplementary Figure 4. Word Clouds for depression, diabetes distress and diabetes burnout. 23](#_Toc79753144)

[References 24](#_Toc79753145)

### Supplementary Table 1. Search strategy

| **Database** | **Query** | |  |
| --- | --- | --- | --- |
| PsychInfo (06-29-2020) Results = 124 | ((diabetes burnout[TW])) OR (diabetes distress[TW])) OR (diabetes depression[TW]) AND Type 1 diabetes[TW]) | |  |
| Updated PsychInfo (06/23/2021) | ((diabetes burnout[TW])) OR (diabetes distress[TW])) OR (diabetes depression[TW]) AND Type 1 diabetes[TW]) Filters: 2020-2021 | |  |
| PubMed (06-12-2020)  Results = 2323 | (((((((Diabetes Mellitus [MH] OR Diabetes[TW] OR Diabetic[TW]) AND ((Stress, Psychological [MH] OR Stress[TW] OR Distress[TW] ) OR (Depression[MH] OR Depressive[TW] OR Depression[TW]) OR (Burnout, Psychological[MH] OR Burnout[TW]))) NOT ("ER stress"[TW] OR "cold stress"[TW] OR "cytokine stress"[TW] OR "endogenous stress"[TW] OR "Exercise Heat Stress"[TW] OR "Hemodynamic Stress"[TW] OR "inflammatory stress"[TW] OR "metabolic stress"[TW] OR "mineral stress"[TW] OR "MGO stress"[TW] OR "oxidative stress"[TW] OR "reticulum stress"[TW] OR "postoperative stress"[TW] OR "shear stress"[TW] OR "stress hyperglycaemia"[TW] OR "Stress-Induced Lipid"[TW] OR "Stress Mechanical"[TW] OR "respiratory distress"[TW])) AND (("Type 1 diabetes"[TW]) OR ("Type 2 diabetes"[TW]))) OR ((("diabetes burnout"[TW] OR "diabetes distress"[TW] OR "diabetes-related distress"[TW] OR "diabetes depression"[TW]) AND ("Type 1 diabetes"[TW])) OR (("diabetes burnout"[TW] OR "diabetes distress"[TW] OR "diabetes-related distress"[TW] OR "diabetes depression"[TW]) AND ("Type 2 diabetes"[TW])))) NOT (Gestational[TW])) NOT( Ketoacidosis [TW])) AND ("Type 1 diabetes"[TW]) Filters: English, Humans | |  |
| Updated PubMed (06-17-2021) | (((((((Diabetes Mellitus [MH] OR Diabetes[TW] OR Diabetic[TW]) AND ((Stress, Psychological [MH] OR Stress[TW] OR Distress[TW] ) OR (Depression[MH] OR Depressive[TW] OR Depression[TW]) OR (Burnout, Psychological[MH] OR Burnout[TW]))) NOT ("ER stress"[TW] OR "cold stress"[TW] OR "cytokine stress"[TW] OR "endogenous stress"[TW] OR "Exercise Heat Stress"[TW] OR "Hemodynamic Stress"[TW] OR "inflammatory stress"[TW] OR "metabolic stress"[TW] OR "mineral stress"[TW] OR "MGO stress"[TW] OR "oxidative stress"[TW] OR "reticulum stress"[TW] OR "postoperative stress"[TW] OR "shear stress"[TW] OR "stress hyperglycaemia"[TW] OR "Stress-Induced Lipid"[TW] OR "Stress Mechanical"[TW] OR "respiratory distress"[TW])) AND (("Type 1 diabetes"[TW]) OR ("Type 2 diabetes"[TW]))) OR ((("diabetes burnout"[TW] OR "diabetes distress"[TW] OR "diabetes-related distress"[TW] OR "diabetes depression"[TW]) AND ("Type 1 diabetes"[TW])) OR (("diabetes burnout"[TW] OR "diabetes distress"[TW] OR "diabetes-related distress"[TW] OR "diabetes depression"[TW]) AND ("Type 2 diabetes"[TW])))) NOT (Gestational[TW])) NOT( Ketoacidosis [TW])) AND ("Type 1 diabetes"[TW]) Filters: English, Humans AND 2020-2021 | |  |
| Web of Science (07-24-2020) Results = 1927 | ((((((Ts=(Diabetes OR Diabetic) ) AND (Ts=(Stress OR Distress OR Depression OR Depressive OR Burnout) ) AND (Ts="Type 1 diabetes") )) OR (Ts=("diabetes burnout" OR "diabetes distress" OR "diabetes-related distress" OR "diabetes depression") AND (Ts="Type 1 diabetes") )) NOT (Ts=("ER stress" OR "cold stress" OR "cytokine stress" OR "endogenous stress" OR "Exercise Heat Stress" OR "Hemodynamic Stress" OR "inflammatory stress" OR "metabolic stress" OR "mineral stress" OR "MGO stress" OR "oxidative stress" OR "reticulum stress" OR "postoperative stress" OR "shear stress" OR "stress hyperglycaemia" OR "Stress-Induced Lipid" OR "Stress Mechanical" OR "respiratory distress") )) NOT (Ts=(Gestational OR Ketoacidosis) )) AND LANGUAGE: (English)  Indexes=SCI-EXPANDED, SSCI, A&HCI, CPCI-S, CPCI-SSH, ESCI Timespan=All years | |  |
| Updated Web of Science (06/29/2021) | ((((((Ts=(Diabetes OR Diabetic) ) AND (Ts=(Stress OR Distress OR Depression OR Depressive OR Burnout) ) AND (Ts="Type 1 diabetes") )) OR (Ts=("diabetes burnout" OR "diabetes distress" OR "diabetes-related distress" OR "diabetes depression") AND (Ts="Type 1 diabetes") )) NOT (Ts=("ER stress" OR "cold stress" OR "cytokine stress" OR "endogenous stress" OR "Exercise Heat Stress" OR "Hemodynamic Stress" OR "inflammatory stress" OR "metabolic stress" OR "mineral stress" OR "MGO stress" OR "oxidative stress" OR "reticulum stress" OR "postoperative stress" OR "shear stress" OR "stress hyperglycaemia" OR "Stress-Induced Lipid" OR "Stress Mechanical" OR "respiratory distress") )) NOT (Ts=(Gestational OR Ketoacidosis) )) AND LANGUAGE: (English)  Indexes=SCI-EXPANDED, SSCI, A&HCI, CPCI-S, CPCI-SSH, ESCI Timespan=2020-2021 | |  |
| **Database** | | **Query** | |
| PsychInfo (06-29-2020) Results = 124 | | ((diabetes burnout[TW])) OR (diabetes distress[TW])) OR (diabetes depression[TW]) AND Type 1 diabetes[TW]) | |
| Updated PsychInfo (06/23/2021) | | ((diabetes burnout[TW])) OR (diabetes distress[TW])) OR (diabetes depression[TW]) AND Type 1 diabetes[TW]) Filters: 2020-2021 | |
| PubMed (06-12-2020)  Results = 2323 | | (((((((Diabetes Mellitus [MH] OR Diabetes[TW] OR Diabetic[TW]) AND ((Stress, Psychological [MH] OR Stress[TW] OR Distress[TW] ) OR (Depression[MH] OR Depressive[TW] OR Depression[TW]) OR (Burnout, Psychological[MH] OR Burnout[TW]))) NOT ("ER stress"[TW] OR "cold stress"[TW] OR "cytokine stress"[TW] OR "endogenous stress"[TW] OR "Exercise Heat Stress"[TW] OR "Hemodynamic Stress"[TW] OR "inflammatory stress"[TW] OR "metabolic stress"[TW] OR "mineral stress"[TW] OR "MGO stress"[TW] OR "oxidative stress"[TW] OR "reticulum stress"[TW] OR "postoperative stress"[TW] OR "shear stress"[TW] OR "stress hyperglycaemia"[TW] OR "Stress-Induced Lipid"[TW] OR "Stress Mechanical"[TW] OR "respiratory distress"[TW])) AND (("Type 1 diabetes"[TW]) OR ("Type 2 diabetes"[TW]))) OR ((("diabetes burnout"[TW] OR "diabetes distress"[TW] OR "diabetes-related distress"[TW] OR "diabetes depression"[TW]) AND ("Type 1 diabetes"[TW])) OR (("diabetes burnout"[TW] OR "diabetes distress"[TW] OR "diabetes-related distress"[TW] OR "diabetes depression"[TW]) AND ("Type 2 diabetes"[TW])))) NOT (Gestational[TW])) NOT( Ketoacidosis [TW])) AND ("Type 1 diabetes"[TW]) Filters: English, Humans | |
| Updated PubMed (06-17-2021) | | (((((((Diabetes Mellitus [MH] OR Diabetes[TW] OR Diabetic[TW]) AND ((Stress, Psychological [MH] OR Stress[TW] OR Distress[TW] ) OR (Depression[MH] OR Depressive[TW] OR Depression[TW]) OR (Burnout, Psychological[MH] OR Burnout[TW]))) NOT ("ER stress"[TW] OR "cold stress"[TW] OR "cytokine stress"[TW] OR "endogenous stress"[TW] OR "Exercise Heat Stress"[TW] OR "Hemodynamic Stress"[TW] OR "inflammatory stress"[TW] OR "metabolic stress"[TW] OR "mineral stress"[TW] OR "MGO stress"[TW] OR "oxidative stress"[TW] OR "reticulum stress"[TW] OR "postoperative stress"[TW] OR "shear stress"[TW] OR "stress hyperglycaemia"[TW] OR "Stress-Induced Lipid"[TW] OR "Stress Mechanical"[TW] OR "respiratory distress"[TW])) AND (("Type 1 diabetes"[TW]) OR ("Type 2 diabetes"[TW]))) OR ((("diabetes burnout"[TW] OR "diabetes distress"[TW] OR "diabetes-related distress"[TW] OR "diabetes depression"[TW]) AND ("Type 1 diabetes"[TW])) OR (("diabetes burnout"[TW] OR "diabetes distress"[TW] OR "diabetes-related distress"[TW] OR "diabetes depression"[TW]) AND ("Type 2 diabetes"[TW])))) NOT (Gestational[TW])) NOT( Ketoacidosis [TW])) AND ("Type 1 diabetes"[TW]) Filters: English, Humans AND 2020-2021 | |
| Web of Science (07-24-2020) Results = 1927 | | ((((((Ts=(Diabetes OR Diabetic) ) AND (Ts=(Stress OR Distress OR Depression OR Depressive OR Burnout) ) AND (Ts="Type 1 diabetes") )) OR (Ts=("diabetes burnout" OR "diabetes distress" OR "diabetes-related distress" OR "diabetes depression") AND (Ts="Type 1 diabetes") )) NOT (Ts=("ER stress" OR "cold stress" OR "cytokine stress" OR "endogenous stress" OR "Exercise Heat Stress" OR "Hemodynamic Stress" OR "inflammatory stress" OR "metabolic stress" OR "mineral stress" OR "MGO stress" OR "oxidative stress" OR "reticulum stress" OR "postoperative stress" OR "shear stress" OR "stress hyperglycaemia" OR "Stress-Induced Lipid" OR "Stress Mechanical" OR "respiratory distress") )) NOT (Ts=(Gestational OR Ketoacidosis) )) AND LANGUAGE: (English)  Indexes=SCI-EXPANDED, SSCI, A&HCI, CPCI-S, CPCI-SSH, ESCI Timespan=All years | |
| Updated Web of Science (06/29/2021) | | ((((((Ts=(Diabetes OR Diabetic) ) AND (Ts=(Stress OR Distress OR Depression OR Depressive OR Burnout) ) AND (Ts="Type 1 diabetes") )) OR (Ts=("diabetes burnout" OR "diabetes distress" OR "diabetes-related distress" OR "diabetes depression") AND (Ts="Type 1 diabetes") )) NOT (Ts=("ER stress" OR "cold stress" OR "cytokine stress" OR "endogenous stress" OR "Exercise Heat Stress" OR "Hemodynamic Stress" OR "inflammatory stress" OR "metabolic stress" OR "mineral stress" OR "MGO stress" OR "oxidative stress" OR "reticulum stress" OR "postoperative stress" OR "shear stress" OR "stress hyperglycaemia" OR "Stress-Induced Lipid" OR "Stress Mechanical" OR "respiratory distress") )) NOT (Ts=(Gestational OR Ketoacidosis) )) AND LANGUAGE: (English)  Indexes=SCI-EXPANDED, SSCI, A&HCI, CPCI-S, CPCI-SSH, ESCI Timespan=2020-2021 | |

### Supplementary Table 2. Inclusion criteria

|  | **Inclusion Criteria** | **Exclusion Criteria** |
| --- | --- | --- |
| Population | Type 1 Diabetes | Only type 2 diabetes, do not differentiate between type 1 and type 2 diabetes |
| Concept | Depression  Diabetes Distress  Diabetes Burnout |  |
| Context | All Settings |  |
| Method | Original research | Reviews  Editorials  Case- Reports  Conference Reports  Study Protocols |
| Year of Publication | 01/1990- 07/2020 | Published before 1990 |
| Language | English Language | Other Language |

### **Supplementary Table 3**. Summary of all studies

| **First Author, Year,** | **Country** | **Sample size** | **Aim** | **Study Design** | **Age group** | **Context** | **Tool Depression** | **Tool Distress** | **Tool Burnout** |
| --- | --- | --- | --- | --- | --- | --- | --- | --- | --- |
| **reference** |  |  |  |  |  |  |  |  |  |
| **Studies where depression was the main concept** | | | | | | | | | |
| Abo, 2020^1^ | Egypt | 100 | To assess depression in adolescents with T1D and the impact of obesity and diabetes complications on depression | CRS | ADO | OUTP | PHQ | - | - |
| Adal, 2015^2^ | Turkey | 295 | To determine the psychosocial aspects of Type 1 diabetes | CRS | ADO | OUTP | CDI | - | - |
| Ahola, 2020^3^ | Finland | 1046 | To study the association between depressive symptoms and markers of health in T1D | CRS | ADU | DBA | BDI | - | - |
| Ahola, 2021^4^ | Finland | 1339 | To assess the association between depressive symptoms and leisure-time physical activity in T1D | CRS | ADU | DBA | BDI | - | - |
| Aschner, 2021^5^ | Colombia | 2280 | To assess the prevalence and risk factors for depressive symptoms in patients with diabetes | CRS | ADU | OUTP | PHQ | - | - |
| Baran, 2018^6^ | Turkey | 141 | To observe the psychological characteristics of type 1 diabetes with children | CRS | CHI & ADO | OUTP | CDI | - | - |
| Basli, 2020^7^ | Turkey | 21 | To observe the effects of active expressionist art therapy techniques on depression, anxiety, quality of life and the management of diabetes in adolescents | LS | ADO | OUTP | BDI | - | - |
| Baucom, 2015^8^ | US | 175 | To observe the relationship between depressive symptoms in diabetes and adherence with daily stress | LS | ADO | OUTP | CES-D | - | - |
| Bot, 2013^9^ | NL | 158 | To determine the relationship of individual depressive symptoms in diabetes and its effect on glycaemic control | LS | ADU | OUTP | PHQ | - | - |
| Brodar, 2021^10^ | US | 232 | To describe the results of a universal and comprehensive psychosocial screening program in an integrated paediatric diabetes clinic | CRS | ADO | OUTP | PHQ | - | - |
| Castellano-Guerrero, 2018^11^ | Spain | 339 | To determine the prevalence of depression and anxiety in diabetes between genders and ages | CRS | ADU | OUTP | MINI | - | - |
| Castellano-Guerrero, 2020^12^ | Spain | 312 | To assess gender differences in terms of quality of life and socio-demographic, clinical and psychological factors in adults with long-standing T1D | CRS | ADU | OUTP | MINI | - | - |
| Corathers, 2013^13^ | US | 509 | To determine the prevalence of diabetes depression and screening tools to assess depression | CRS | ADO | OUTP | CDI | - | - |
| Corathers, 2019^14^ | US | 1291 | To observe diabetes depression screening tools and approaches to depression over a period of time | LS | ADO | OUTP | CDI | - | - |
| de Groot, 1999^15^ | US | 33 | To observe the relationship between diabetes depression and glycaemic control | LS | ADU | OUTP | DSM-3 | - | - |
| de Wit, 2008^16^ | NL | 91 | To assess the impact of monitoring and discussions in health-related quality of life on psychosocial well-being | RCT | CHI | INP | CES-D | - | - |
| de Wit, 2010^17^ | NL | 81 | To determine the impact of discussion on quality of life and monitoring with adolescents living with diabetes | CRS | ADO | OUTP | CES-D | - | - |
| de Wit, 2011^18^ | NL | 233 | To determine rate of depression and psychological needs | LS | ADO | OUTP | CDI | - | - |
| Duda-Sobczak, 2016^19^ | Poland | 283 | To investigate the prevalence of depressive symptoms among those with longstanding diabetes | CRS | ADU | OUTP | BDI | - | - |
| Eckert, 2021^20^ | Germany | 62438 | To assess the association between thyroid autoimmunity and psychiatric disorders in adolescents and young adults with T1D | CRS | ADO | DBA | DSM-5 | - | - |
| Egbuonu, 2021^21^ | US | 249 | To compare adults with T1D screened for depression with those without seen in a real-world setting | CCS | ADU | OUTP | PHQ | - | - |
| Ehrmann, 2017^22^ | Germany | 604 | To determine the relationship between depression subtypes and glycaemic control | CRS | ADU | INP & OUTP | CES-D | PAID | - |
| Fisher, 2016^23^ | US | 368 | To measure the prevalence of diabetes depression and addressing over diagnosis | CRS | ADU | OUTP | PHQ, DSM-5 | DDS | - |
| Forlani, 2006^24^ | Italy | 90 | To determine the impact of an empowerment based program in well-being and quality of life | LS | ADU | OUTP | PGWB | - | - |
| Forlani , 2013^25^ | Italy | 55 | To determine the impact of a psychological support program | LS | ADU | OUTP | PGWB | - | - |
| Geirhos, 2021^26^ | Germany | 11 | To identify the challenges and coping strategies of adolescents and young adults as well as their needs and preferred intervention characteristics for cognitive-behavioural treatment on the Internet. | QR | ADO & ADU | OUTP | PHQ | - | - |
| Gendelman, 2009^27^ | US | 1004 | To investigate the prevalence of depression and antidepressant medication use among those with and without diabetes | CRS | ADU | GENPOP | BDI | - | - |
| Gilsanz, 2018^28^ | US | 3742 | To observe the relationship between diabetes depression and major glycaemic events | CRS | ADU | GENPOP | ICD | - | - |
| Grey, 1998^29^ | US | 65 | To assess the impact of a behavioural intervention with an intensive management program on glycaemic control and well being | RCT | CHI & ADO | OUTP | CDI | - | - |
| Grey, 2001^30^ | US | 81 | To assess the impact of an intensive management program on glycaemic control and well being | RCT | CHI & ADO | OUTP | CDI | - | - |
| Grey, 2009^31^ | US | 82 | To evaluate the impact of coping skill training program in school-aged children on glycaemic control and well being | RCT | CHI | OUTP | CDI | - | - |
| Guo, 2013^32^ | China | 136 | To determine diabetes management, depressive symptoms, and glycaemic control in youth | CRS | CHI & ADO | OUTP | DSRS | - | - |
| Hamburger, 2020^33^ | US | 120 | To evaluate the association of depressive symptoms and sleep with glycaemic control in adolescents with T1D | CRS | ADO | OUTP | PHQ | - | - |
| Hannonen, 2015^34^ | Finland | 63 | To determine psychological impact of diabetes in children and their mothers | CRS | CHI | OUTP | BASC | - | - |
| Hapunda, 2015^35^ | Zambia | 157 | To determine prevalence of diabetes and its relationship to depression in Zambia | CRS | CHI, ADO & ADU | INP & GENPOP | MDI | - | - |
| Harrington, 2021^36^ | US | 301 | To examine the associations between diabetes self-management, HbA1c, and psychosocial out-comes with the frequency of depressive symptoms. | CRS | ADO | OUTP | CES-D | - | - |
| Hassan, 2006^37^ | US | 222 | To determine how socioeconomic status and depression plays a role on diabetes and glycaemic control | CRS | CHI & ADO | OUTP | CDI | - | - |
| Hendrieckx, 2020^38^ | Australia | 440 | To assess impaired emotional well-being in T1D and T2D | CRS | ADU | OUTP | WHO-5 | PAID | - |
| Hislop, 2008^39^ | Australia | 92 | To assess the prevalence of distress in adolescents living with diabetes | CRS | ADU | OUTP | BDI, CES-D | - | - |
| Hoff, 2002^40^ | US | 68 | To evaluate the relationship between distress, illness uncertainty and diabetes | CRS | CHI & ADO | OUTP | GSI | - | - |
| Hood, 2011^41^ | US | 145 | To observe the impact of diabetes depression on glycaemic control over a period of time | LS | ADO | OUTP | CDI | - | - |
| Insabella, 2007^42^ | US | 117 | To assess the impact of a behavioural intervention on psychological outcomes and glycaemic control | RCT | ADO | OUTP | CDI | - | - |
| Ismail, 2008^43^ | UK | 344 | To assess the impact of motivational enhancement therapy with or without cognitive behaviour therapy on glycaemic control | RCT | ADU | INP | PHQ | - | - |
| Jaser, 2008^44^ | US | 108 | To determine the relationship of diabetes depression between children and their mothers | CRS | CHI | ONL | CDI | - | - |
| Jaser, 2012^45^ | US | 320 | To observe the seasonal trends of diabetes depression | CRS | ADO | GENPOP | CDI | - | - |
| Jones, 2016^46^ | Denmark | 502 | To examine the psychological impact of diabetes | CRS | ADU | OUTP | WHO-5 | PAID | - |
| Karlson, 1997^47^ | Sweden | 155 | To observe the impact of diabetes depression on burden of illness, glycaemic control and its symptoms | CRS | ADU | OUTP | SCL-90 | - | - |
| Khater, 2017^48^ | Egypt | 86 | To assess the frequency of diabetes depression in children | LS | CHI | OUTP | CES-D | - | - |
| Knight, 2015^49^ | US | 50 | To identify risk factors for depression and anxiety in youth | CRS | CHI | OUTP | PHQ | - | - |
| Kohen, 1998^50^ | UK | 100 | To assess the impact of diabetes depression and anxiety in quality of life and symptom reports | CRS | ADU | OUTP | HADS | - | - |
| Korbel, 2007^51^ | US | 127 | To observe gender difference of depressive symptoms in diabetes and its glycaemic outcome and management | CRS | CHI | OUTP | CDI | - | - |
| Kovacs, 1997^52^ | US | 24 | To investigate depression and diabetes and its outcomes | LS | ADO | INP | DSM-3 | - | - |
| Iina, 2021^53^ | Finland | 65 | To assess psychological flexibility and its association with glycaemic control and quality of life in adolescents with diabetes | CRS | ADO | OUTPs | BDI | - | - |
| Littlefield, 1990^54^ | Canada | 158 | To assess social support in diabetes depression and its impact | CRS | ADU | INP | BDI | - | - |
| Lloyd, 2000^55^ | UK | 109 | To determine the prevalence of diabetes depression and anxiety in a clinic | CRS | ADU | OUTP | HADS | - | - |
| Lloyd, 2003^56^ | UK | 208 | To observe the cultural difference between the United Kingdom and the United States in prevalence of diabetes depression and its outcomes | CRS | ADU | OUTP | BDI | - | - |
| Lu, 2017^57^ | Taiwan | 1373 | To determine the incidence of diabetes depression in a female Taiwanese children | LS | CHI & ADO | DBA | ICD | - | - |
| Lustman, 2005^58^ | US | 188 | To analyse the role of self-care in depression related hyperglycaemia | CRS | ADU | OUTP | SCL-90 | - | - |
| Maia, 2014^59^ | Brazil | 110 | To determine the prevalence of depression and anxiety in type 1 diabetes | CRS | ADU | OUTP | WHO-5, HADS | - | - |
| Majidi, 2021^60^ | US | 86 | To examine whether a shared medical appointment model developed specifically for adolescents with T1D will improve psychosocial outcomes | RCT | CHI | OUTP | CDI | - | - |
| Missambou Mandilou, 2021^61^ | Congo | 74 | To describe the psychosocial functioning and assess the associated risk factors of children, adolescents and young adults with T1D. | CRS | ADO & ADU | OUTP & INP | BDI | - | - |
| Marker, 2019^62^ | US | 1009 | To specify the clinic-wide diabetes screening initiative among children with diabetes | LS | ADO | OUTP | PHQ | - | - |
| Massano-Cardoso, 2020^63^ | Portugal | 347 | To examine the differences of glycaemic control among patients with depressive symptoms in type 1 and type 2 diabetes | CRS | ADU | OUTP | BDI | - | - |
| McDade-Montez, 2011^64^ | US | 61 | To investigate the influence of depression on diabetes outcomes | CRS | ADU | OUTP | IDAS, PANAS-X | - | - |
| McGill, 2018^65^ | US | 96 | To determine the effect of depressive symptoms in diabetes outcomes | LS | CHI | OUTP | CDI | - | - |
| McGrady, 2009^66^ | US | 276 | To investigate the role blood glucose monitoring plays in depressive symptoms and glycaemic control | LS | ADO | OUTP | CDI | - | - |
| McGrady, 2010^67^ | US | 144 | To observe depressive symptoms from diabetes in adolescents | CRS | ADO | OUTP | CDI | - | - |
| Melin, 2013^68^ | Sweden | 292 | To observe factors impacting glycaemic control such as depression, obesity and smoking | CRS | ADU | OUTP | HADS | - | - |
| Melin, 2020^69^ | Sweden | 283 | To explore whether sTWEAK was associated with depression | CRS | ADU | OUTP | HADS | - | - |
| Merchant, 2011^70^ | US | 23 | To examine psychosocial outcomes of diabetes in adolescents and its effect on glycaemic control | CRS | CHI & ADO | OUTP | BDI | - | - |
| Morgan, 2014^71^ | UK | 5548 | To determine how if there is a prevalence of diabetes depression in adolescents and compare it with other age groups | LS | ADO & ADU | OUTP | ICD | - | - |
| Munaf, 2016^72^ | Pakistan | 50 | To assess the difference of anxiety and depression among people living with type 1 and type 2 diabetes | CRS | ADU | OUTP | HADS | - | - |
| Nguyen, 2021^73^ | Netherlands | 154 | To examine whether parental depression and anxiety predict adolescent emotional distress and HbA1c | CRS | ADO | OUTP | CDI | - | - |
| Niemcryk, 1990^74^ | US | 48 | To investigate the impact of psychosocial factors on glycaemic control | LS | ADU & ADU | INP & OUTP | CES-D | - | - |
| Nunley, 2019^75^ | US | 67 | To determine the prevalence of depression in diabetes and its relationship to cerebral microvascular disease | CRS | ADU | INP | BDI | - | - |
| Oris, 2016^76^ | Belgium | 197 | To observe the changes of depressive symptoms in adolescents over a period of time | LS | ADU | OUTP | CES-D | PAID | - |
| Peyrot, 1997^77^ | US | 578 | To determine the prevalence of depression and anxiety among people living with diabetes | LS | ADU | OUTP | CES-D | - | - |
| Picozzi, 2019^78^ | US | 156 | To determine the impact of depression among adolescents on glycaemic control | LS | ADO | INP | PHQ | - | - |
| Pouwer, 2010^79^ | NL | 933 | To determine the prevalence of depression among patients living with diabetes from three outpatient clinics | LS | ADU | OUTP | WHO-5, CES-D | - | - |
| Riley, 2015^80^ | US | 82 | To investigate the impact of behavioural family system therapy on depressive symptoms from diabetes | RCT | ADO | DBA | CDI | - | - |
| Rostami, 2016^81^ | Iran | 74 | To examine the impact of group training intervention on anxiety and depressive symptoms among those living with diabetes | RCT | ADO | INP | BDI | - | - |
| Roy 1994^82^ | US | 62 | To understand the association of depressive symptoms in diabetes with recent life events | CRS | ADU | OUTP | BDI | - | - |
| Roy, 2001^83^ | US | 581 | To observe depressive symptoms from diabetes among African-Americans | LS | ADU | INP | BDI | - | - |
| Roy, 2007^84^ | US | 315 | To assess the risk of depressive symptoms in diabetes for retinopathy | LS | ADU | OUTP | BDI | - | - |
| Ruiz-Aranda, 2020^85^ | Spain | 30 | To analyse the associations between resilience, diabetes-related quality of life, and anxiety and depression in T1D patients with fear of hypoglycaemia | CRS | ADU | OUTP | BDI | - | - |
| Schmitt, 2017^86^ | Germany | 430 | To observe the role of diabetes self-management on depressive symptoms and hyperglycaemia | CRS | ADU | INP | CES-D | - | - |
| Schmitt, 2017^87^ | Germany | 181 | To investigate the impact of control of depressive symptoms in diabetes with glycaemic control | CRS | ADU | OUTP | CES-D | - | - |
| Schmitt, 2021^88^ | Germany | 205 | To examine whether symptoms of depression or anxiety predict glycaemia and incident diabetes complications | CRS | ADU | GENPOP | PHQ-8 | - | - |
| Sendela, 2015^89^ | Poland | 477 | To investigate the prevalence of depressive symptoms in diabetes among school-aged children | CRS | CHI | OUTP | CDI | - | - |
| Shaban, 2006^90^ | UK | 273 | To observe the prevalence of diabetes and anxiety in people living with diabetes | CRS | AD & ADU | OUTP | HADS | - | - |
| Silverstein, 2015^91^ | US | 261 | To evaluate the prevalence of depressive symptoms in diabetes and management | CRS | CHI | OUTP | CDI | - | - |
| Spiess, 1995^92^ | Austria | 23 | To determine the impact of an intervention on glycaemic control and psychosocial well-being | LS | ADU | INP | BDI | - | - |
| Stewart, 2005^93^ | US | 231 | To observe the relationship between depressive symptoms in diabetes and rates of hospitalizations | CRS | ADO | OUTP | CES-D | - | - |
| Stewart, 2011^94^ | US | 219 | To observe the relationship between somatic items on the depression inventory and cognitive symptoms in youth living with diabetes | RCT | ADO | OUTP | CES-D | - | - |
| Tittel, 2021^95^ | Germany | 79067 | To analyse coeliac disease and depression associations in children, adolescents, and young adults with T1D | CRS | CHI, ADO & ADU | DBA | DSM-5 | - | - |
| van der Ven, 2005^96^ | NL | 107 | To determine the short-term impact of cognitive behavioural group training (CBGT) on glycaemic control and psychosocial well-being | RCT | ADU | OUTP | CES-D | - | - |
| Van Tilburg, 2001^97^ | US | 64 | Investigate the association between depressed mood and glycaemic control | LS | ADU | OUTP | BDI | - | - |
| Vlahou, 2021^98^ | Qatar | 150 | To identify culturally appropriate psychological screening measures for children and adolescents with T1D in Qatar | CRS | CHI & ADO | OUTP | MFQ | - | - |
| Wei, 2018^99^ | UK | 85 | To compare the impact of cognitive behavioural therapy with nondirective supportive counselling on glycaemic control and psychological well-being in adolescents with type 1 diabetes | RCT | CHI | INP | WBQ | - | - |
| Williams, 2009^100^ | US | 187 | To examine the association of demographic factors, diabetes variables and psychological distress in parents, children and adolescents with family conflicts | LS | CHI & ADO | OUTP | CDI | - | - |
| Wisting, 2020^101^ | Norway | 282 | To investigate correlates of eating disorder psychopathology among adults with T1D | CRS | ADU | OUTPs | HAD | - | - |
| Wolfgram, 2020^102^ | US | 1225 | Validation of the PHQ9 for teens | LS | ADO | OUTP | PHQ | - | - |
| Wu, 2013^103^ | US | 150 | To examine the relationships between parental involvement and depressive symptoms in adolescents on blood sugar monitoring and glycaemic control | LS | ADO | INP | CDI | - | - |
| Yayan, 2019^104^ | Turkey | 88 | To assess quality of life and its association with depression in young people with T1D | CRS | ADO | INP | CDI | - | - |
| Zaffani, 2015^105^ | Italy | 214 | To study the prevalence of anxiety, depression and quality of life in young patients with T1D and in controls and its correlation with glycaemic control | LS | CHI | INP & GENPOP | CDI | - | - |
| Zdunczyk, 2014^106^ | Poland | 372 | To assess the prevalence of depressive symptoms in T1D children treated with an insulin pump. | LS | CHI | OUTP | CDI | - | - |
| **Studies where diabetes distress is the main concept** | | | | | | | | | |
| Adams, 2018^107^ | US | 29 | To determine the psychosocial impact of a Hybrid Closed Loop system | LS | ADO & ADU | OUTP | - | DDS | - |
| Al Hayek, 2020^108^ | Saudi Arabia | 187 | To study and explore the intervention of the flash glucose monitoring system on diabetes-related distress in children and adolescents with T1D | LS | ADO | OUTP | - | T1-DDS | - |
| Balfe, 2013^109^ | Ireland | 35 | To investigate factors that lead to diabetes distress | QR | ADU | OUTP | - | - | - |
| Beverly, 2019^110^ | US | 85 | To measure the prevalence of diabetes distress among university students | CRS | ADU | OUTP | - | DDS | - |
| Cechetti, 2020^111^ | Brazil | 100 | To analyse the association between emotional distress and HbA1c in adolescents and young adults with T1D | CRS | ADO & ADU | OUTP | - | DDS | - |
| d’Emden, 2017^112^ | Australia | 151 | To describe psychosocial characteristics and management of diabetes in young people | LS | ADU | OUTP | - | PAID | - |
| Danne, 2021^113^ | Germany | 1575 | To evaluate diabetes distress in a cohort of adults with T1D receiving sotagliflozin to optimized insulin | RCT | ADU | OUTP | - | DDS | - |
| do Nascimento Andrade, 2019^114^ | Brazil | 68 | To evaluate the impact of socioeconomic and psychological factors on diabetes management | CRS | CHI | OUTP |  | PAID |  |
| Ebrahimpour, 2015^115^ | Iran | 30 | To assess the impact of an interactive computer game on diabetes distress among children | RCT | CHI | INP | - | OSBD–R | - |
| Evans, 2019^116^ | US | 804 | To provide a standardized guide of care for parents and children with diabetes distress | CRS | CHI | OUTP | - | PAID | - |
| Fegan-Bohm, 2020^117^ | US | 187 | To assess the association of lower SES and elevated HbA1c with higher levels of diabetes-related distress | CRS | CHI & ADO | OUTP | - | PAID | - |
| Fisher, 2015^118^ | US | 414 | To investigate the factors leading to diabetes distress | LS | ADU | OUTP | PHQ | DDS | - |
| Fisher, 2016^119^ | US | 224 | To determine the prevalence, incidence and stability over time of diabetes distress | CRS | ADU | OUTP | - | DDS | - |
| Fisher, 2018^120^ | US | 301 | To evaluate the effective of two interventions aimed to reduce diabetes distress | CRS | ADU | OUTP | - | DDS | - |
| Fisher, 2018^121^ | US | 301 | To establish how emotion regulation leads to diabetes distress | RCT | ADU | OUTP & GENPOP | PHQ | DDS | - |
| Griva, 2020^122^ | Greece | 102 | To examine the factor structure of T1-DDS in the Greek population and to assess its psychometric properties for use in research and clinical practice | CRS | ADU | OUTP | - | T1-DDS | - |
| Hansen, 2019^123^ | Denmark | 1030 | To evaluate how work-related factors play a role in diabetes distress | CRS | ADU | OUTP | - | PAID | - |
| Hansen, 2020^124^ | Denmark | 1594 | To investigate the relationship between diabetes stigma experienced by adults with T1D and diabetes outcomes using the new validated measure of the T1D Stigma Rating Scale | CRS | ADU | Online | - | PAID | - |
| Al Hayek, 2020^125^ | Saudi Arabia | 95 | To evaluate the association of FreeStyle Libre in diabetes distress and sleep quality | LS | ADU | OUTP | - | DDS | - |
| Hessler, 2017^126^ | US | 305 | To investigate the long-term effects of diabetes distress on management | CRS & LS | ADU | OUTP | PHQ | DDS | - |
| Hessler, 2020^127^ | US | 102 | To compare the effectiveness of two interventions aimed to reduce diabetes distress | LS | ADU | GENPOP | - | DDS | - |
| Iturralde, 2017^128^ | US | 264 | To test the association between avoidant coping and diabetes distress | RCT | ADO | INP | - | PAID | - |
| Iturralde, 2019^129^ | US | 264 | To observe the impact an intervention on diabetes distress in adolescents over a period of time | RCT | ADO | OUTP | - | PAID | - |
| Joensen, 2020^130^ | Denmark | 471 | To map specific COVID-19 concerns and overall psychosocial health among people with diabetes in the initial phase of the COVID-19 pandemic in Denmark | CRS | ADU | Online | - | DDS | - |
| Kelly, 2020^131^ | UK | 199 | To assess if anxious and avoidant attachments are associated with diabetes distress | CRS | ADU | OUTPs | - | DDS | - |
| Khan, 2018^132^ | UK | 129 | To investigate the impact of self-management behaviours on diabetes distress | CRS | ADU | OUTP | - | DDS | - |
| Knight, 2017^133^ | Austria | 21 | To determine the impact of a novel education program aimed to improve psychosocial well-being | RCT | ADU | INP | - | PAID | - |
| Kuniss, 2019^134^ | Germany | 26 | To assess the impact of diabetes treatment and teaching program in diabetes distress | LS | ADU | OUTP | - | PAID | - |
| Lašaite, 2016^135^ | Lithuania | 538 | To observe the differences in diabetes distress between male and females in adolescents and young adults | CRS | ADO & ADU | OUTP | - | PAID | - |
| Lašaite, 2016^136^ | Lithuania | 214 | To observe the difference of diabetes distress between genders and age of Type 1 diabetes onset | CRS | ADU | OUTP | - | DDS | - |
| Law, 2013^137^ | UK | 213 | To investigate the consequences of diabetes distress in children and parents | CRS | ADO | OUTP | - | PAID | - |
| Li, 2009^138^ | US | 713 | To observe the risk factors associated with diabetes and psychological distress | CRS | ADU | GENPOP | - | K6 | - |
| Lee, 2020^139^ | Taiwan | 177 | To model pathways from diabetes distress, peer feedback, parenting style, and self-management to HbA1c levels in adolescents with T1D | CRS | ADO | INP | - | PAID | - |
| Lohiya, 2021^140^ | India | 67 | To assess the diabetes-specific distress perceived by children and adolescents with T1D and their mothers | CRS | CHI & ADO | OUTP | - | PAID | - |
| Luo, 2021^141^ | China | 100 | To describe domains and associated factors of diabetes distress | CRS | ADO | OUTP | - | DDS | - |
| Messer, 2020^142^ | US | 282 | To explore predictors of consistent CGM use in adolescents with T1D | CRS | ADO | Online | - | PAID | - |
| Miyawaki, 2015^143^ | Japan | 11 | To investigate the differences in distress among people diagnosed with different types of diabetes | CRS | ADU | INP | - | PAID | - |
| Nouwen, 2009^144^ | UK | 151 | To compare self-efficacy and illness beliefs in adolescents with diabetes distress | CRS | ADO | OUTP | - | PAID | - |
| Oldham, 2020^145^ | UK | 54 | To assess the effect of continuous subcutaneous insulin infusion on glycaemic control, hypoglycaemia, and emotional distress in adults with T1D | LS | ADU | Diabetes Clinic | - | PAID | - |
| Polonsky, 1995^146^ | US | 70 | Describe the development of the PAID, a new questionnaire for evaluating diabetes distress | CRS | ADU | OUTP | - | PAID | - |
| Polonsky, 2016^147^ | US | 1269 | To investigate the impact of the Omnipod (R) intervention with Quality of Life | CRS | ADU | GENPOP | - | DDS | - |
| Polonsky, 2017^148^ | US | 155 | To explore the impact of Continuous Glucose Monitoring intervention on Quality of Life | RCT | ADU | OUTP | - | DDS | - |
| Polonsky, 2021^149^ | US | 302 | To examine experiences of sharing real-time blood glucose monitoring data and its impact on health-related outcomes | CRS | ADU | OUTP | - | T1-DDS | - |
| Powers, 2017^150^ | US | 274 | To explore the psychological behaviours associated with diabetes distress | CRS | ADO & ADU | OUTP | - | DDS | - |
| Schmitt , 2016^151^ | Germany | 628 | To delineate diabetes distress and depression and its association to glycaemic control | CRS | ADU | OUTP | CES-D | PAID, DDS | - |
| Snoek, 2000^152^ | NL | 1472 | To observe the cultural difference between the United States and Dutch populations of the validity of the PAID scale among people living with diabetes | PPD | ADU | GENPOP | - | PAID | - |
| Snoek, 2001^153^ | US | 24 | To determine the impact of cognitive behavioural group therapy intervention of glycaemic control and management | CRS | ADU | OUTP | - | PAID | - |
| Spaic, 2019^154^ | Canada | 205 | To observe the transition of care in young adults and its effects on glycaemic control and diabetes distress | RCT | ADO & ADU | OUTP | - | DDS | - |
| Speight, 2016^155^ | Australia | 506 | To determine the impact of a program on diabetes distress and its management benefits | QE | ADU | OUTP | - | PAID | - |
| Stahl-Pehe, 2019^156^ | Germany | 584 | To evaluate the impact of diabetes distress in young adults on glycaemic control and health status | LS | ADU | GENPOP | PHQ | PAID |  |
| Stanulewicz, 2019^157^ | UK | 1547 | To assess the validity of the PAID-11 scale | CRS | ADU | OUTP | HADS | PAID | - |
| Strandberg, 2015^158^ | Norway | 175 | To follow-up on the impact of diabetes distress on glycaemic control | LS | ADU | OUTP | - | DDS | - |
| Todd, 2018^159^ | UK | 280 | To observe the relationship between hypoglycaemia, glycaemic control and diabetes distress | CRS | ADU | OUTP | - | DDS | - |
| van Beers, 2017^160^ | NL | 52 | To assess the impact of Continuous Glucose Monitoring on diabetes distress | RCT | ADU | OUTP | WHO-5 | PAID | - |
| Vesco, 2018^161^ | US | 1040 | Compare the type of technology used on diabetes-related distress and HbA1c in adolescents | LS | CHI & ADO | OUTP | - | PAID | - |
| Vesco, 2018^162^ | US | 906 | To examine the influence of the concordance in reports of distress between adolescents with T1D and glycaemic control | LS | ADO | OUTP | - | PAID | - |
| Weissberg-Benchell, 2011^163^ | US | 130 | Adapting the PAID for use with adolescents with type 1 diabetes | RCT | ADO | INP | - | PAID | - |
| Weissberg-Benchell, 2019^164^ | US | 1978 | To assess associations between camp attendance, diabetes distress, and perceived autonomy in diabetes self-management | LS | CHI & ADO | INP | - | PAID | - |
| Wilmot, 2021^165^ | UK | 3858 | To assess the association between glycaemic control and patient-reported outcomes in adults with T1D | CRS | ADU | OUTP | - | PAID | - |
| **Studies where diabetes burnout is the main concept** | | | | | | | | | |
| Abdoli, 2019^166^ | US | 18 | To address experiences related to diabetes burnout | QR | ADU | OUTP | - | - | - |
| Abdoli, 2019^167^ | US | 21 | To address the experiences of people living with diabetes burnout | QR | NA | ONL | - | - | MBI-GS |
| Abdoli , 2020^168^ | US | 32 | To evaluate self-reported accounts of diabetes burnout using YouTube | QR | NA | ONL | - | - | - |
| Helgeson, 2021^169^ | US | 88 | To develop a measure of diabetes burnout and to link this measure to psychological health and diabetes outcomes | CRS | ADU | INP | - | - | - |
| Lowes, 2015^170^ | UK | 693 | Perspectives of type 1 children and adolescents on managing diabetes | CRS | CHI & ADO | OUTP | - | - | - |
| **Studies were depression and diabetes distress were the main concepts** | | | | | | | | | |
| Blanchette, 2021^171^ | US | 413 | To describe the relationships between financial stressors and psychological factors on self-management outcomes in emerging adults with T1D | CRS | ADU | OUTP | CES-D | T1-DDS | - |
| Due-Christensen, 2012^172^ | Denmark | 54 | To determine the impact of diabetes support groups psychologically and in glycaemic control | MM | ADU | OUTP | GSI, SCL-90 | PAID | - |
| Fleer, 2013^173^ | NL | 166 | To assess diabetes depression and distress by screening in a clinic | CRS | ADU | OUTP | CES-D | PAID | - |
| Hagger, 2018^174^ | Australia | 450 | To examine the association of diabetes distress and depression to glycaemic outcomes | CRS | ADO | OUTP | PHQ | PAID | - |
| Hapunda, 2020^175^ | Zambia | 93 | To determine the prevalence of fear of hypoglycaemia in patients with diabetes | CRS | ADO & ADU | OUTP | MDI | PAID | - |
| Holmes-Truscott, 2020^176^ | Australia | 959 | To evaluate the association of diabetes stigma with psychological and hba1c outcomes | CRS | ADU | OUTP | PHQ | PAID | - |
| Hood, 2018^177^ | US | 264 | To evaluate the effectiveness of a program in diabetes distress and depression | RCT | CHI & ADO | OUTP | CDI | PAID | - |
| Hopkins , 2012^178^ | UK | 639 | To assess the impact of an education programme | RCT | ADU | OUTP | HADS | PAID | - |
| Kampling, 2018^179^ | Germany | 299 | To investigate how diabetes depression, anxiety and distress impacts over a period of time with glycaemic control | LS | ADU | INP | SCL-90 | FBD | - |
| Liu, 2020^180^ | US | 494 | To study factors associated with the behavioural and emotional aspects of fear of hypoglycaemia in adults living with T1D | CRS | ADU | Online | PHQ | PAID | - |
| McIntyre, 2010^181^ | Australia | 145 | To describe the impact of the Dose adjustment for normal eating intervention on glycaemic control | RCT | ADU | OUTP | HADS | PAID | - |
| Nguyen, 2021^182^ | Netherlands | 171 | To assess anxiety and depression in adolescents with T1D, and its association with parental emotional distress | CRS | ADO | OUTP | CDI | PAID | - |
| Raymaekers, 2020^183^ | Belgium | 559 | To identify trajectory classes of perceived peer functioning in youth with T1D, based on peer support and extreme peer orientation | CRS | ADO & ADU | OUTP | CES-D | PAID | - |
| Raymaekers, 2021^184^ | Belgium | 324 | To examine the association of satisfaction with support from friends over diabetes management in adults with T1D | CRS | ADO | INP | CES-D | PAID | - |
| Schmitt, 2015^185^ | Germany | 466 | To delineate diabetes distress and depression and its association to glycaemic control | CRS | ADU | OUTP | CES-D | DDS | - |
| Schmitt, 2020^186^ | Germany | 339 | To analyse the independent associations of depression and diabetes distress with self- management and glycaemic outcome in T1D & T2D | CRS | ADU | OUTP | PHQ-9 | PAID-5 | - |
| Silveria, 2019^187^ | Brazil | 63 | To observe empowerment and diabetes distress in low income individuals and its impact on glycaemic control | CRS | ADU | OUTP | HADS, DSM-5 | DDS | - |
| Snoek, 2008^188^ | NL | 86 | To compare the effectiveness of two interventions aimed to reduce depressive symptoms | RCT | ADU | INP | CES-D | PAID | - |
| Strandberg, 2014^189^ | Norway | 319 | To investigate diabetes distress, depression and anxiety and its association to glycaemic control | CRS | ADU | OUTP | HADS | PAID, DDS | - |
| Tanenbaum, 2012^190^ | US | 34 | To determine the influence of a clinician's assessment of depression on diabetes distress | CRS | ADU | INP | CES-D | DDS | - |
| Weissberg-Benchell, 2016^191^ | US | 264 | To compare the efficacy of prevention of depression and resilience with an advanced diabetes education program | LS | ADO | OUTP | CDI | PAID | - |
| Weissberg-Benchell, 2020^192^ | US | 264 | To analyse the rate and shape of change of outcomes from pre-intervention across post-intervention and follow-up time points | RCT | ADO | OUTP | CDI | PAID-T | - |
| Wong , 2020^193^ | US | 60 | To study the psychosocial effects of a monetary strengthening intervention on self-monitoring of blood glucose in young people with T1D. | RCT | ADO | OUTP | HADS | PAID | - |
| Wong , 2021^194^ | US | 124 | To discover acceptability profiles and assess demographic and clinical correlates in adolescents with diabetes | CRS | ADO | OUTP | PHQ | DDS | - |
| Younes, 2021^195^ | AE | 72 | To assess the prevalence of diabetes-related distress, depression, and their association with glycaemic control in adolescents with T1D | CRS | ADO | OUTP | CDI | PAID | - |
| Zajdel, 2021^196^ | US | 199 | To determine whether assessment of partner's illness interacts with individual self-efficacy to predict health outcomes | CRS | ADU | OUTP | CES-D | DDS | - |
| Zhu, 2020^197^ | Singapore | 98 | To assess the association between psychological state and blood glucose at baseline and 2 years after transition in a cohort of young adults with diabetes | CRS | ADO & ADU | OUTP | HADS | PAID | - |
| **Studies were burnout, diabetes distress and depression were the main concepts** | | | | | | | | | |
| Abdoli , 2020^198^ | US | 111 | To demonstrate the initial data pertaining to diabetes burnout, distress and depression and its associations | CRS | ADU | OUTP | PHQ-8 | DDS-T1 | MBI-GS, IIQ, MATCH, DES-SF |
| Abdoli , 2020^199^ | US | 31 | To study dimensions of diabetes burnout and to compare with diabetes distress and depressive symptoms among adults with T1D | CRS | ADU | OUTP | - | - | - |
| Abdoli , 2021^200^ | US | 1099 | To assess reliability and validity of the Diabetes Burnout Scale among adults with T1D | CRS | ADU | SMED & ONL | PHQ | T1-DDS | DBS |
| Abdoli , 2021^201^ | US | 1788 | To examine psychosocial well-being and diabetes outcomes in people with T1D during the COVID-19 pandemic | CRS | ADU | ONL | PHQ | T1-DDS | DBS |
| Abbreviations: AE: United Arab Emirates; NL: The Netherlands; UK: United Kingdom; US: United States; T1D: type 1 diabetes; T2D: type 2 diabetes; CRS: cross-sectional study; MM, mixed methods; QE: Quasi-experimental; QR: Qualitative research; QE: Quasi-experimental; RCT: Randomized controlled trial; LS: Observational longitudinal study; PPD: pre-post design; ADO: Adolescents; ADU: Adults; CHI: Children; OUTP: Outpatients; DBA: Database from cohort or registry; INP: inpatients; GENPOP: General population; ONL: Online; SMED: Social media; CDI: Children’s Depression Inventory; CES-D: Center for Epidemiological Studies Depression Scale; BDI: Beck Depression Inventory; DSM: Diagnostic and Statistical Manual of Mental Disorders; HADS: Hospital Anxiety and Depression Scale; PHQ: Patient Health Questionnaire; ICD: International Classification of Diseases codes; SCL-90-R: Hopkins Symptom Checklist; WHO-5: World Health Organisation- Five Well-Being Index; PGWB: Psychological General Well-Being Index; GSI: Global Severity Index; MDI: Major Depression Inventory; DSRS: Depression Self-Rating Scale of Children; BASC: Behavior Assessment System for Children; PANAS-X: Positive and Negative Affect Schedule - Expanded Form; IDAS: Inventory of Depression and Anxiety Symptoms; WBQ: Well-Being Questionnaire ; MINI: Mini International Neuropsychiatric Interview; MFQ: Mood and Feelings Questionnaire; PAID: Problem Areas in Diabetes Questionnaire; DDS: Diabetes Distress Scale; OSBD–R: Observational Scale of Behavioral Distress–Revised; K6: Kessler Psychological Distress; FBD: Fragebogen zu Alltagsbelastungen bei Diabetes; MBI-GS: Maslach Burnout Inventory-General Survey; IIQ: Illness Identity Questionnaire; MATCH: Motivation and Attitude Toward Changing Health; DES-SF: Diabetes Empowerment Scale-Short Form; DBS: Diabetes Burnout Scale | | | | | | | | | |

### Supplementary Table 4. Frequency and overlapping of sub concepts: depression, diabetes distress and diabetes burnout

| **Sub concept** | **Frequency in depression** | **Frequency in distress** | **Frequency in burnout** |
| --- | --- | --- | --- |
| **Depression sub concepts without overlapping** | | | |
| Change in sleep | 61 | 0 | 0 |
| Loss of appetite | 52 | 0 | 0 |
| Loss of interest | 42 | 0 | 0 |
| Hopelessness | 38 | 0 | 0 |
| Suicidal Ideation | 32 | 0 | 0 |
| Tearfulness | 29 | 0 | 0 |
| Disappointment in one self | 28 | 0 | 0 |
| Ineffectiveness | 21 | 0 | 0 |
| Poor self-esteem | 21 | 0 | 0 |
| Agitation | 20 | 0 | 0 |
| Anhedonia | 20 | 0 | 0 |
| Interpersonal problems | 18 | 0 | 0 |
| Could not get "going" | 16 | 0 | 0 |
| Weight loss | 14 | 0 | 0 |
| Poor compliance to treatment | 12 | 0 | 0 |
| Dissatisfaction | 11 | 0 | 0 |
| Anxiety | 8 | 0 | 0 |
| Difficulties diabetes/self-management | 7 | 0 | 0 |
| Feeling slowed down | 5 | 0 | 0 |
| Dysphoric | 4 | 0 | 0 |
| Poor glycaemic control | 4 | 0 | 0 |
| Pessimism | 3 | 0 | 0 |
| **Distress sub concepts without overlapping** | | | |
| Fear of hypoglycemia | 0 | 55 | 0 |
| Physician distress | 0 | 53 | 0 |
| Worries about long-term complications | 0 | 48 | 0 |
| Uncertainty of diabetes care goals | 0 | 39 | 0 |
| Denial | 0 | 38 | 0 |
| Discouraged with treatment plan | 0 | 38 | 0 |
| Feeling deprived of food | 0 | 38 | 0 |
| Uncomfortable in social settings | 0 | 37 | 0 |
| Management distress | 0 | 33 | 0 |
| Family/friend distress | 0 | 19 | 0 |
| Worry | 0 | 19 | 0 |
| Negative social perception | 0 | 17 | 0 |
| Can't control eating | 0 | 15 | 0 |
| Emotional burden | 0 | 15 | 0 |
| Over-vigilance | 0 | 6 | 0 |
| Lack of confidence in self-care | 0 | 4 | 0 |
| Need for emotional support | 0 | 3 | 0 |
| **Burnout sub concepts without overlapping** | | | |
| Exhaustion | 0 | 0 | 89 |
| Detachment from diabetes care | 0 | 0 | 67 |
| Detachment from illness identity | 0 | 0 | 56 |
| Apathy | 0 | 0 | 33 |
| Detachment from self | 0 | 0 | 11 |
| Lack of achievement | 0 | 0 | 11 |
| **Overlapping depression and distress sub concepts** | | | |
| Negative mood | 60 | 48 | 0 |
| Poor concentration | 45 | 2 | 0 |
| Loss of energy | 38 | 38 | 0 |
| Fear | 23 | 39 | 0 |
| Lonely | 18 | 37 | 0 |
| Feeling of failure | 17 | 8 | 0 |
| Feeling guilty | 3 | 39 | 0 |
| **Overlapping distress and burnout sub concepts** | | | |
| Burned out | 0 | 33 | 37 |
| Powerlessness to manage diabetes | 0 | 15 | 67 |
| Frustration | 0 | 8 | 11 |
| Overwhelmed | 0 | 44 | 11 |
| Anger | 0 | 33 | 40 |
| **Overlapping depression, distress and burnout sub concepts** | | | |
| Detachment from support systems | 30 | 40 | 78 |

###

### Supplementary Figure 1. Number of publications by regions of the world

Bar length represents the number of publications by region


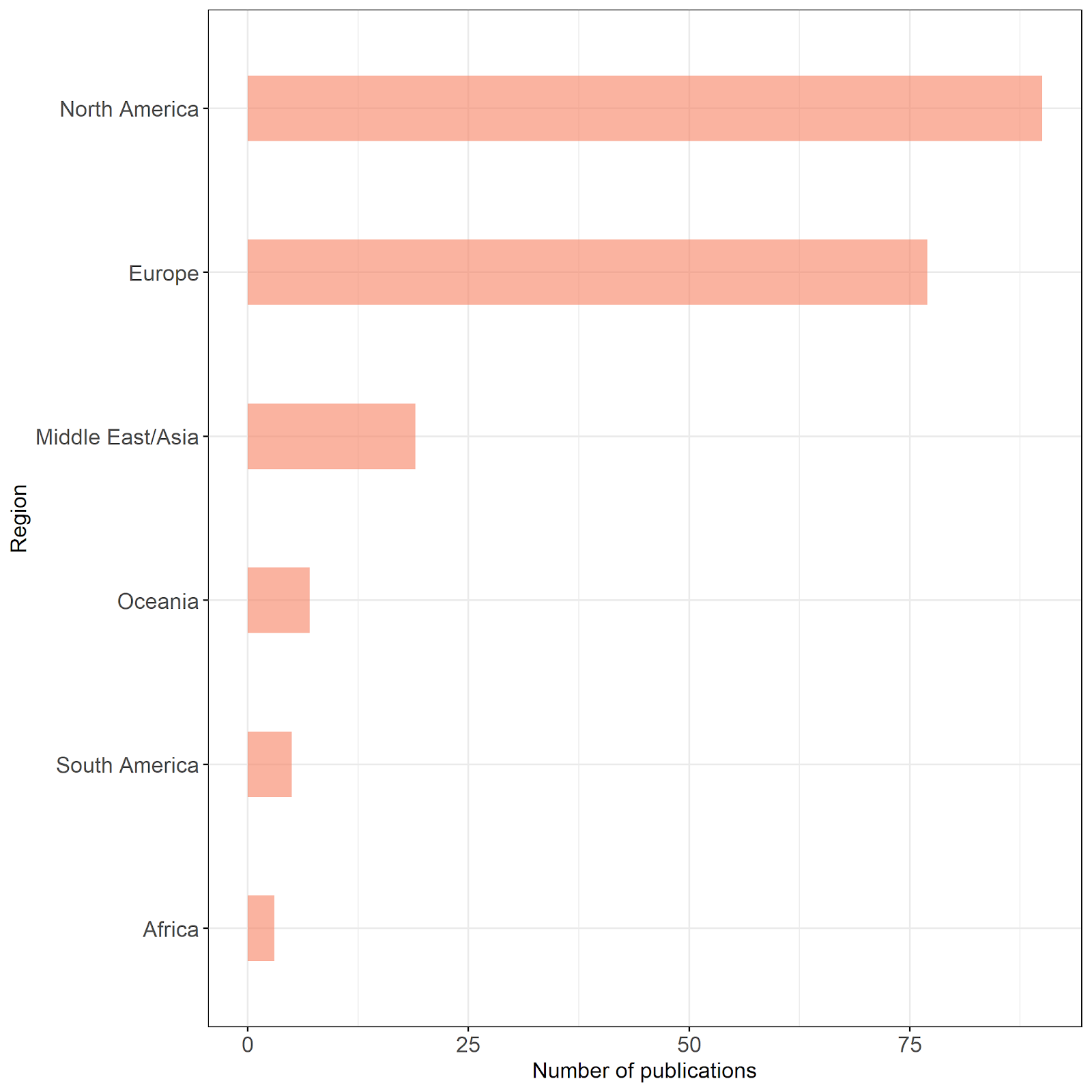


### **Supplementary Figure 2.** Circular bar plot of concepts of depression, diabetes distress and diabetes burnout by authors’ descriptions

Bar length represents the frequency of words or phrases for each concept in percentage among the 201 included studies. Words or phrases found in studies about depression, diabetes distress and diabetes burnout are in blue, green and salmon respectively. Abbreviations: Detach.from sup.system: Detachment from support system


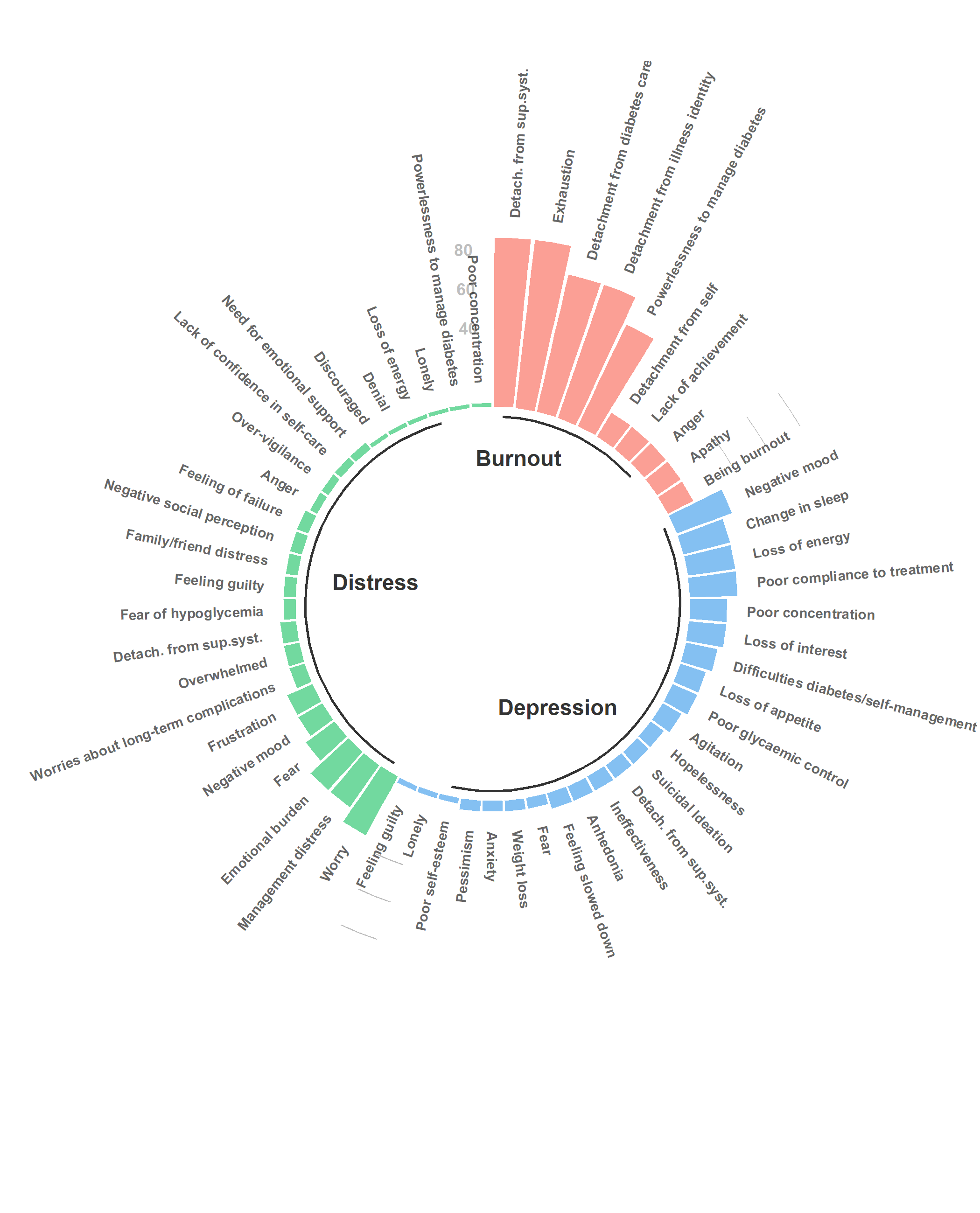


### **Supplementary Figure 3.** Circular bar plot of concepts of depression, diabetes distress and diabetes burnout by questionnaire items

Bar length represents the frequency of words or phrases for each concept in percentage among the 201 included studies. Words or phrases found in studies about depression, diabetes distress and diabetes burnout are in blue, green and salmon respectively. Abbreviations: Detach.from sup.system: Detachment from support system; Detach. from.illness id.: Detachment from illness identification; Uncomfort.social: Uncomfortable in social settings


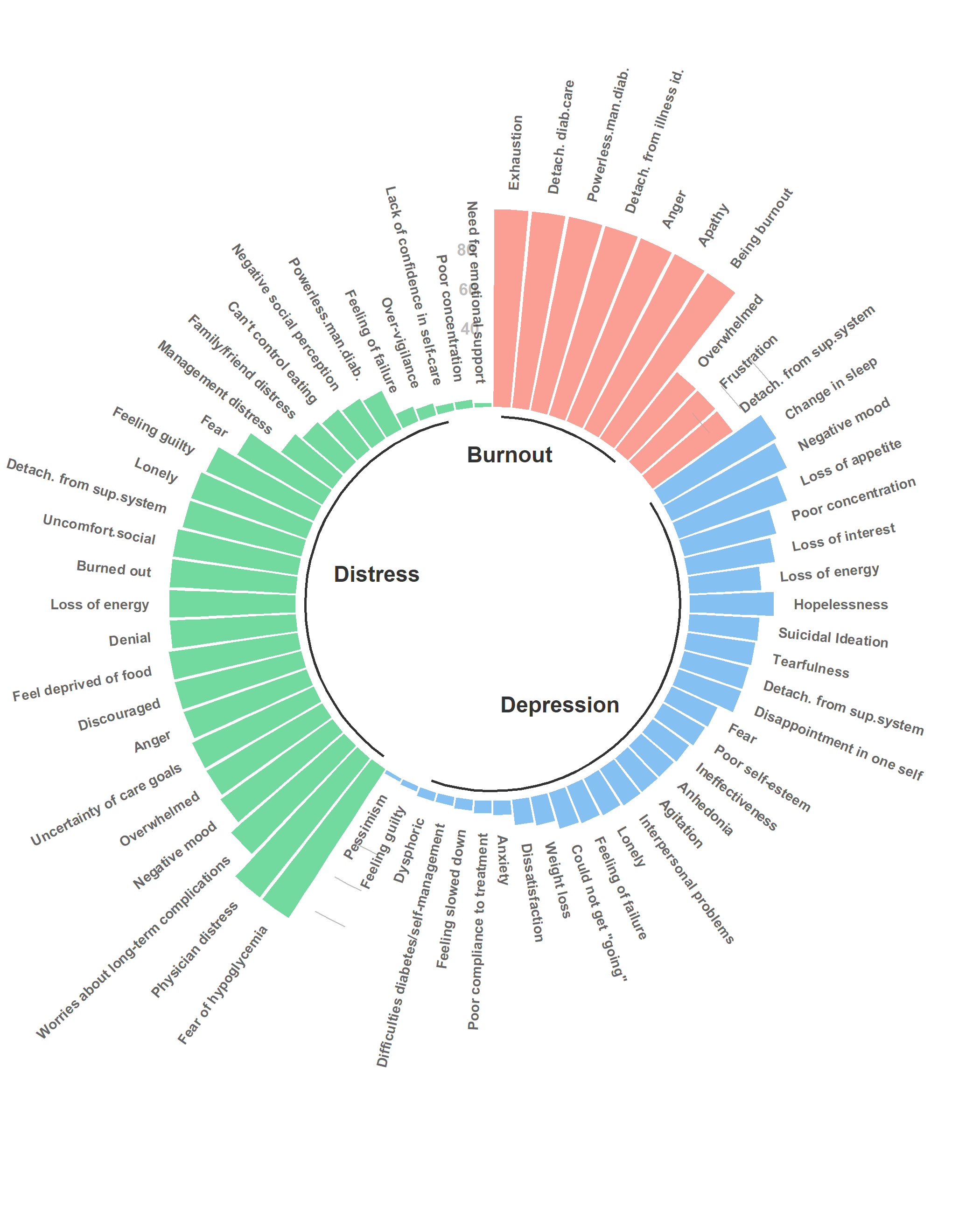


### Supplementary Figure 4. Word Clouds for depression, diabetes distress and diabetes burnout.

Word size reflects how often the word was cited among the studies included by each concept. Words with an equivalent citation frequency share the same colour.
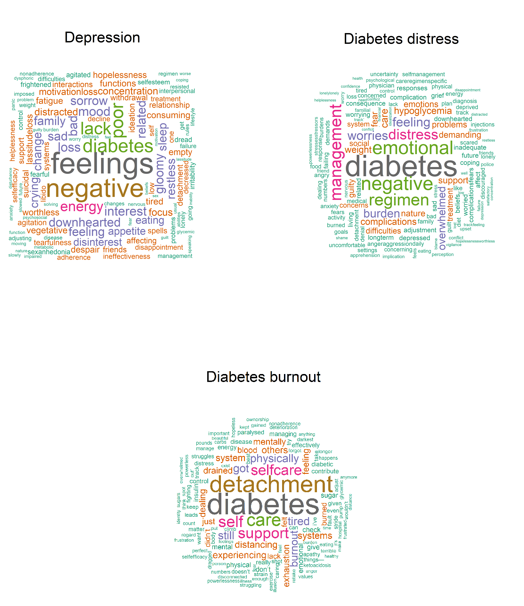


### References

1. Abo Elasrar M, Hany Elrassas H, Adel Thabet R, Seifeldin Abdeen M, Eldeen Nouby Mohamed Elazab A. Obesity and diabetic control as predictors for depression in adolescents with type 1 diabetes mellitus. *Vulnerable Children and Youth Studies* 2020: 1-14.

2. Adal E, Önal Z, Ersen A, Yalçın K, Önal H, Aydın A. Recognizing the psychosocial aspects of type 1 diabetes in adolescents. *Journal of clinical research in pediatric endocrinology* 2015; **7**(1): 57.

3. Ahola AJ, Radzeviciene L, Zaharenko L, et al. Association between symptoms of depression, diabetes complications and vascular risk factors in four European cohorts of individuals with type 1 diabetes - InterDiane Consortium. *Diabetes Res Clin Pract* 2020; **170**: 108495.

4. Ahola AJ, Tikkanen-Dolenc H, Forsblom C, Harjutsalo V, Groop P-H. Symptoms of depression are associated with reduced leisure-time physical activity in adult individuals with type 1 diabetes. *Acta diabetologica* 2021: 1-8.

5. Aschner P, Gagliardino JJ, Ilkova H, et al. High prevalence of depressive symptoms in patients with type 1 and type 2 diabetes in developing countries: results from the International Diabetes Management Practices Study. *Diabetes Care* 2021; 44(5): 1100-7.

6. Baran RT, Surer-Adanir A, Karakurt MN, et al. Evaluation of psychological characteristics of Turkish children with type 1 diabetes mellitus from two demographically and geographically distinct regions. 2018.

7. Basli E, Ozmen S, Demirci E, Kendirci M, Tatli ZU, Kondolot M. The Effects of Art Therapy Techniques on Depression, Anxiety Levels and Quality of Life in the Adolescent with Type 1 Diabetes Mellitus: A Preliminary Study. *Erciyes Medical Journal* 2020; **42**(4): 431-6.

8. Baucom KJ, Queen TL, Wiebe DJ, et al. Depressive symptoms, daily stress, and adherence in late adolescents with type 1 diabetes. *Health Psychology* 2015; **34**(5): 522.

9. Bot M, Pouwer F, De Jonge P, Tack C, Geelhoed‐Duijvestijn P, Snoek FJ. Differential associations between depressive symptoms and glycaemic control in outpatients with diabetes. *Diabetic medicine* 2013; **30**(3): e115-e22.

10. Brodar KE, Davis EM, Lynn C, et al. Comprehensive psychosocial screening in a pediatric diabetes clinic. *Pediatric diabetes* 2021; **22**(4): 656-66.

11. Castellano-Guerrero A, Guerrero R, Relimpio F, et al. Prevalence and predictors of depression and anxiety in adult patients with type 1 diabetes in tertiary care setting. *Acta diabetologica* 2018; **55**(9): 943-53.

12. Castellano-Guerrero AM, Guerrero R, Ruiz-Aranda D, et al. Gender differences in quality of life in adults with long-standing type 1 diabetes mellitus. *Diabetology & Metabolic Syndrome* 2020; **12**(1): 1-7.

13. Corathers SD, Kichler J, Jones N-HY, et al. Improving depression screening for adolescents with type 1 diabetes. *Pediatrics* 2013; **132**(5): e1395-e402.

14. Corathers S, Mara CA, Chundi PK, Kichler JC. Depression Screening of Adolescents With Diabetes: 5-Years of Implementation and Outcomes. *Journal of the American Academy of Child and Adolescent Psychiatry* 2019; **58**(6): 628-32.

15. de Groot M, Jacobson AM, Samson JA, Welch G. Glycemic control and major depression in patients with type 1 and type 2 diabetes mellitus. *Journal of psychosomatic research* 1999; **46**(5): 425-35.

16. De Wit M, Delemarre-van de Waal HA, Bokma JA, et al. Monitoring and discussing health-related quality of life in adolescents with type 1 diabetes improve psychosocial well-being: a randomized controlled trial. *Diabetes care* 2008; **31**(8): 1521-6.

17. De Wit M, Delemarre‐van de Waal HA, Bokma JA, et al. Follow‐up results on monitoring and discussing health‐related quality of life in adolescent diabetes care: benefits do not sustain in routine practice. *Pediatric diabetes* 2010; **11**(3): 175-81.

18. de Wit M, Snoek FJ. Depressive symptoms and unmet psychological needs of Dutch youth with type 1 diabetes: results of a web‐survey. *Pediatric diabetes* 2011; **12**(3pt1): 172-6.

19. Duda-Sobczak A, Zozulińska-Ziółkiewicz D, Wierusz-Wysocka B. Prevalence of depressive symptoms and diagnosed depression among subjects with longstanding type 1 diabetes and no serious chronic complications, hospitalized due to inadequate metabolic control of diabetes. *Clinical Diabetology* 2016; **5**(6): 173-7.

20. Eckert A, Galler A, Papsch M, et al. Are psychiatric disorders associated with thyroid hormone therapy in adolescents and young adults with type 1 diabetes? *Journal of diabetes* 2021.

21. Egbuonu I, Trief PM, Roe C, Weinstock RS. Glycemic outcomes related to depression in adults with type 1 diabetes. *J Health Psychol* 2021; **26**(6): 786-94.

22. Ehrmann D, Schmitt A, Reimer A, Haak T, Kulzer B, Hermanns N. The affective and somatic side of depression: subtypes of depressive symptoms show diametrically opposed associations with glycemic control in people with type 1 diabetes. *Acta diabetologica* 2017; **54**(8): 749-56.

23. Fisher L, Hessler DM, Polonsky WH, et al. Prevalence of depression in Type 1 diabetes and the problem of over-diagnosis. *Diabetic medicine : a journal of the British Diabetic Association* 2016; **33**(11): 1590-7.

24. Forlani G, Zannoni C, Tarrini G, Melchionda N, Marchesini G. An empowerment-based educational program improves psychological well-being and health-related quality of life in Type 1 diabetes. *Journal of endocrinological investigation* 2006; **29**(5): 405-12.

25. Forlani G, Nuccitelli C, Caselli C, et al. A psychological support program for individuals with Type 1 diabetes. *Acta diabetologica* 2013; **50**(2): 209-16.

26. Geirhos A, Lunkenheimer F, Holl RW, et al. Involving patients' perspective in the development of an internet-and mobile-based CBT intervention for adolescents with chronic medical conditions: Findings from a qualitative study. *Internet interventions* 2021; **24**: 100383.

27. Gendelman N, Snell-Bergeon JK, McFann K, et al. Prevalence and correlates of depression in individuals with and without type 1 diabetes. *Diabetes care* 2009; **32**(4): 575-9.

28. Gilsanz P, Karter AJ, Beeri MS, Quesenberry CP, Whitmer RA. The bidirectional association between depression and severe hypoglycemic and hyperglycemic events in type 1 diabetes. *Diabetes Care* 2018; **41**(3): 446-52.

29. Grey M, Boland EA, Davidson M, Yu C, Sullivan-Bolyai S, Tamborlane WV. Short-term effects of coping skills training as adjunct to intensive therapy in adolescents. *Diabetes care* 1998; **21**(6): 902-8.

30. Grey M, Davidson M, Boland EA, Tamborlane WV. Clinical and psychosocial factors associated with achievement of treatment goals in adolescents with diabetes mellitus. *Journal of adolescent health* 2001; **28**(5): 377-85.

31. Grey M, Whittemore R, Jaser S, et al. Effects of coping skills training in school‐age children with type 1 diabetes. *Research in nursing & health* 2009; **32**(4): 405-18.

32. Guo J, Whittemore R, Grey M, Wang J, Zhou ZG, He GP. Diabetes self‐management, depressive symptoms, quality of life and metabolic control in youth with type 1 diabetes in China. *Journal of clinical nursing* 2013; **22**(1-2): 69-79.

33. Hamburger ER, Goethals ER, Choudhary A, Jaser SS. Sleep and depressive symptoms in adolescents with type 1 diabetes not meeting glycemic targets. *Diabetes Res Clin Pract* 2020; **169**: 108442.

34. Hannonen R, Eklund K, Tolvanen A, et al. Psychological distress of children with early‐onset type 1 diabetes and their mothers' well‐being. *Acta Paediatrica* 2015; **104**(11): 1144-9.

35. Hapunda G, Abubakar A, Pouwer F, van de Vijver F. Diabetes mellitus and comorbid depression in Zambia. *Diabetic Medicine* 2015; **32**(6): 814-8.

36. Harrington KR, Shapira A, Volkening LK, et al. Associations of diabetes self-management characteristics, HbA1c, and psychosocial outcomes with depressive symptoms in a contemporary sample of adolescents with type 1 diabetes. *Journal of diabetes and its complications* 2021; **35**(3): 107838.

37. Hassan K, Loar R, Anderson BJ, Heptulla RA. The role of socioeconomic status, depression, quality of life, and glycemic control in type 1 diabetes mellitus. *The Journal of pediatrics* 2006; **149**(4): 526-31.

38. Hendrieckx C, Halliday JA, Russell-Green S, et al. Adults with diabetes distress often want to talk with their health professionals about it: Findings from an audit of 4 Australian specialist diabetes clinics. *Canadian journal of diabetes* 2020; **44**(6): 473-80.

39. Hislop A, Fegan P, Schlaeppi M, Duck M, Yeap B. Prevalence and associations of psychological distress in young adults with Type 1 diabetes. *Diabetic Medicine* 2008; **25**(1): 91-6.

40. Hoff AL, Mullins LL, Chaney JM, Hartman VL, Domek D. Illness uncertainty, perceived control, and psychological distress among adolescents with type 1 diabetes. *Research and Theory for Nursing Practice* 2002; **16**(4): 223-36.

41. Hood KK, Rausch JR, Dolan LM. Depressive symptoms predict change in glycemic control in adolescents with type 1 diabetes: rates, magnitude, and moderators of change. *Pediatric diabetes* 2011; **12**(8): 718-23.

42. Insabella G, Grey M, Knafl G, Tamborlane W. The transition to young adulthood in youth with type 1 diabetes on intensive treatment. *Pediatric diabetes* 2007; **8**(4): 228-34.

43. Ismail K, Thomas SM, Maissi E, et al. Motivational enhancement therapy with and without cognitive behavior therapy to treat type 1 diabetes: a randomized trial. *Annals of internal medicine* 2008; **149**(10): 708-19.

44. Jaser SS, Whittemore R, Ambrosino JM, Lindemann E, Grey M. Mediators of depressive symptoms in children with type 1 diabetes and their mothers. *Journal of pediatric psychology* 2008; **33**(5): 509-19.

45. Jaser SS, Dumser S, Liberti L, et al. Seasonal trends in depressive symptoms in adolescents with type 1 diabetes. *Diabetes research and clinical practice* 2012; **96**(2): e33-e5.

46. Jones A, Olsen MZ, Perrild HJ, Willaing I. The psychological impact of living with diabetes: Descriptive findings from the DAWN2 study in Denmark. *Primary care diabetes* 2016; **10**(1): 83-6.

47. Karlson B, Agardh CD. Burden of illness, metabolic control, and complications in relation to depressive symptoms in IDDM patients. *Diabetic Medicine* 1997; **14**(12): 1066-72.

48. Khater D, Omar M. Frequency and risk factors of depression in type 1 diabetes in a developing country. *J Pediatr Endocrinol Metab* 2017; **30**(9): 917-22.

49. Knight A, Weiss P, Morales K, et al. Identifying differences in risk factors for depression and anxiety in pediatric chronic disease: a matched cross-sectional study of youth with lupus/mixed connective tissue disease and their peers with diabetes. *The Journal of pediatrics* 2015; **167**(6): 1397-403. e1.

50. Kohen D, Burgess A, Catalan J, Lant A. The role of anxiety and depression in quality of life and symptom reporting in people with diabetes mellitus. *Quality of Life Research* 1998; **7**(3): 197-204.

51. Korbel CD, Wiebe DJ, Berg CA, Palmer DL. Gender differences in adherence to type 1 diabetes management across adolescence: The mediating role of depression. *Children's Healthcare* 2007; **36**(1): 83-98.

52. Kovacs M, Obrosky DS, Goldston D, Drash A. Major depressive disorder in youths with IDDM: a controlled prospective study of course and outcome. *Diabetes care* 1997; **20**(1): 45-51.

53. Iina A, Mirka J, Laura J, Joona M, Raimo L. Adolescents with poorly controlled type 1 diabetes: Psychological flexibility is associated with the glycemic control, quality of life and depressive symptoms. *Journal of Contextual Behavioral Science* 2021; **19**: 50-6.

54. Littlefield CH, Rodin GM, Murray MA, Craven JL. Influence of functional impairment and social support on depressive symptoms in persons with diabetes. *Health Psychology* 1990; **9**(6): 737.

55. Lloyd C, Dyer P, Barnett A. Prevalence of symptoms of depression and anxiety in a diabetes clinic population. *Diabetic medicine* 2000; **17**(3): 198-202.

56. Lloyd CE, Zgibor J, Wilson RR, Barnett AH, Dyer PH, Orchard TJ. Cross-cultural comparisons of anxiety and depression in adults with type 1 diabetes. *Diabetes/metabolism research and reviews* 2003; **19**(5): 401-7.

57. Lu M-C, Juan C-Y, Koo M, Lai N-S. Higher incidence of psychiatrist-diagnosed depression in Taiwanese female school-age children and adolescents with type 1 diabetes: a nationwide, population-based, retrospective cohort study. *Journal of child and adolescent psychopharmacology* 2017; **27**(3): 281-4.

58. Lustman PJ, Clouse RE, Ciechanowski PS, Hirsch IB, Freedland KE. Depression-related hyperglycemia in type 1 diabetes: a mediational approach. *Psychosomatic medicine* 2005; **67**(2): 195-9.

59. Maia AC, Braga Ade A, Paes F, Machado S, Nardi AE, Silva AC. Psychiatric comorbidity in diabetes type 1: a cross-sectional observational study. *Rev Assoc Med Bras (1992)* 2014; **60**(1): 59-62.

60. Majidi S, Reid MW, Fogel J, et al. Psychosocial outcomes in young adolescents with type 1 diabetes participating in shared medical appointments. *Pediatric Diabetes* 2021.

61. Missambou Mandilou SV, Atipo‐Ibara Ollandzobo LC, Kitemo Mpolo FLG, et al. Psychosocial functioning and health related quality of life in children, adolescents and young adults with type 1 diabetes mellitus in Congo. *Pediatric Diabetes* 2021; **22**(4): 675-82.

62. Marker AM, Patton SR, McDonough RJ, Feingold H, Simon L, Clements MA. Implementing clinic‐wide depression screening for pediatric diabetes: An initiative to improve healthcare processes. *Pediatric diabetes* 2019; **20**(7): 964-73.

63. Massano-Cardoso I, Daniel F, Rodrigues V, Galhardo A. Depressive symptoms in Type 1 and Type 2 Diabetes Mellitus and its relationship with glycemic control. 2020.

64. McDade-Montez EA, Watson D. Examining the potential influence of diabetes on depression and anxiety symptoms via multiple sample confirmatory factor analysis. *Annals of Behavioral Medicine* 2011; **42**(3): 341-51.

65. McGill DE, Volkening LK, Pober DM, Muir AB, Young-Hyman DL, Laffel LM. Depressive symptoms at critical times in youth with type 1 diabetes: following type 1 diabetes diagnosis and insulin pump initiation. *Journal of Adolescent Health* 2018; **62**(2): 219-25.

66. McGrady ME, Laffel L, Drotar D, Repaske D, Hood KK. Depressive symptoms and glycemic control in adolescents with type 1 diabetes: mediational role of blood glucose monitoring. *Diabetes care* 2009; **32**(5): 804-6.

67. McGrady ME, Hood KK. Depressive symptoms in adolescents with type 1 diabetes: associations with longitudinal outcomes. *Diabetes research and clinical practice* 2010; **88**(3): e35-e7.

68. Melin EO, Thunander M, Svensson R, Landin-Olsson M, Thulesius HO. Depression, obesity, and smoking were independently associated with inadequate glycemic control in patients with type 1 diabetes. *European journal of endocrinology* 2013; **168**(6): 861-9.

69. Melin EO, Dereke J, Hillman M. Low levels of soluble TWEAK, indicating on-going inflammation, were associated with depression in type 1 diabetes: a cross-sectional study. *BMC psychiatry* 2020; **20**(1): 574.

70. Merchant BR. Type 1 Diabetes in Adolescence: the Effect of Depression, Psychosocial Adjustment, and Duration of Illness on Glycemic Control: Oklahoma State University; 2011.

71. Morgan E, Patterson C, Cardwell C. General practice‐recorded depression and antidepressant use in young people with newly diagnosed Type 1 diabetes: a cohort study using the Clinical Practice Research Datalink. *Diabetic medicine* 2014; **31**(2): 241-5.

72. Munaf S, Asmat A. Anxiety and depression: A study of diabetic types. *FWU Journal of Social Sciences* 2016; **10**(1): 88.

73. Nguyen LA, Pouwer F, Lodder P, et al. Depression and anxiety in adolescents with type 1 diabetes and their parents. *Pediatr Res* 2021: 1-9.

74. Niemcryk SJ, Speers MA, Travis LB, Gary HE. Psychosocial correlates of hemoglobin A1c in young adults with type I diabetes. *Journal of psychosomatic research* 1990; **34**(6): 617-27.

75. Nunley K, Karp J, Orchard T, et al. Depressive symptoms and cerebral microvascular disease in adults with Type 1 diabetes mellitus. *Diabetic Medicine* 2019; **36**(9): 1168-75.

76. Oris L, Luyckx K, Rassart J, et al. Change and stability in depressive symptoms in young adults with type 1 diabetes. *Diabetes research and clinical practice* 2016; **111**: 93-6.

77. Peyrot M, Rubin RR. Levels and risks of depression and anxiety symptomatology among diabetic adults. *Diabetes care* 1997; **20**(4): 585-90.

78. Picozzi A, DeLuca F. Depression and glycemic control in adolescent diabetics: evaluating possible association between depression and hemoglobin A1c. *Public health* 2019; **170**: 32-7.

79. Pouwer F, Geelhoed‐Duijvestijn P, Tack C, et al. Prevalence of comorbid depression is high in out‐patients with Type 1 or Type 2 diabetes mellitus. Results from three out‐patient clinics in the Netherlands. *Diabetic Medicine* 2010; **27**(2): 217-24.

80. Riley AR, Duke DC, Freeman KA, Hood KK, Harris MA. Depressive symptoms in a trial behavioral family systems therapy for diabetes: A post hoc analysis of change. *Diabetes Care* 2015; **38**(8): 1435-40.

81. Rostami S, Naseri M, Dashtbozorgi B, Zarea K, Riaahi Qhahfarrokhi K, Haghighizadeh MH. Effects of Group Training on Depression and Anxiety among Patients with Type I Diabetes: a Randomized Clinical Trial. *International Journal of Pediatrics* 2016; **4**(5): 1777-86.

82. Roy M, Collier B, Roy A. Excess of depressive symptoms and life events among diabetics. *Comprehensive psychiatry* 1994; **35**(2): 129-31.

83. Roy A, Roy M. Depressive symptoms in African‐American type 1 diabetics. *Depression and anxiety* 2001; **13**(1): 28-31.

84. Roy MS, Roy A, Affouf M. Depression is a risk factor for poor glycemic control and retinopathy in African-Americans with type 1 diabetes. *Psychosomatic medicine* 2007; **69**(6): 537-42.

85. Ruiz-Aranda D, Mateo-Rodriguez C, Olmedo IS, García CG, Enríquez AJ, Martinez-Brocca MA. Relationship between Resilience and Quality of Life in Patients with Fear of Hypoglycemia: The Mediating Effects of Anxiety and Depression. *Sustainability* 2020; **12**(20): 8512.

86. Schmitt A, Reimer A, Hermanns N, et al. Depression is linked to hyperglycaemia via suboptimal diabetes self-management: A cross-sectional mediation analysis. *Journal of psychosomatic research* 2017; **94**: 17-23.

87. Schmitt A, Reimer A, Ehrmann D, Kulzer B, Haak T, Hermanns N. Reduction of depressive symptoms predicts improved glycaemic control: secondary results from the DIAMOS study. *Journal of diabetes and its complications* 2017; **31**(11): 1608-13.

88. Schmitt A, McSharry J, Speight J, et al. Symptoms of depression and anxiety in adults with type 1 diabetes: Associations with self-care behaviour, glycaemia and incident complications over four years - Results from diabetes MILES-Australia. *Journal of affective disorders* 2021; **282**: 803-11.

89. Sendela J, Zdunczyk B, Trippenbach-Dulska H, Szypowska A. Prevalence of depressive symptoms in school aged children with type 1 diabetes–A questionnaire study. *Psychiatr Pol* 2015; **49**(5): 1005-16.

90. Shaban M, Fosbury J, Kerr D, Cavan D. The prevalence of depression and anxiety in adults with type 1 diabetes. *Diabetic Medicine* 2006; **23**(12): 1381-4.

91. Silverstein J, Cheng P, Ruedy KJ, et al. Depressive symptoms in youth with type 1 or type 2 diabetes: results of the pediatric diabetes consortium screening assessment of depression in diabetes study. *Diabetes Care* 2015; **38**(12): 2341-3.

92. Spiess K, Sachs G, Pietschmann P, Prager R. A program to reduce onset distress in unselected type I diabetic patients: effects on psychological variables and metabolic control. *European journal of endocrinology* 1995; **132**(5): 580-6.

93. Stewart SM, Rao U, Emslie GJ, Klein D, White PC. Depressive symptoms predict hospitalization for adolescents with type 1 diabetes mellitus. *Pediatrics* 2005; **115**(5): 1315-9.

94. Stewart SM, Simmons A, White PC. Somatic items in the assessment of depressive symptoms in pediatric patients with diabetes. *Journal of behavioral medicine* 2011; **34**(2): 112-9.

95. Tittel SR, Dunstheimer D, Hilgard D, et al. Coeliac disease is associated with depression in children and young adults with type 1 diabetes: results from a multicentre diabetes registry. *Acta diabetologica* 2021; **58**(5): 623-31.

96. Van Der Ven NC, Hogenelst M, Tromp‐Wever A, et al. Short‐term effects of cognitive behavioural group training (CBGT) in adult Type 1 diabetes patients in prolonged poor glycaemic control. A randomized controlled trial. *Diabetic Medicine* 2005; **22**(11): 1619-23.

97. Van Tilburg MA, McCaskill CC, Lane JD, et al. Depressed mood is a factor in glycemic control in type 1 diabetes. *Psychosomatic medicine* 2001; **63**(4): 551-5.

98. Vlahou CH, Petrovski G, Korayem M, et al. Outpatient clinic‐wide psychological screening for children and adolescents with type 1 diabetes in Qatar: An initiative for integrative healthcare in the Gulf region. *Pediatric diabetes* 2021; **22**(4): 667-74.

99. Wei C, Allen RJ, Tallis PM, et al. Cognitive behavioural therapy stabilises glycaemic control in adolescents with type 1 diabetes—Outcomes from a randomised control trial. *Pediatric diabetes* 2018; **19**(1): 106-13.

100. Williams L, Laffel L, Hood K. Diabetes‐specific family conflict and psychological distress in paediatric Type 1 diabetes. *Diabetic Medicine* 2009; **26**(9): 908-14.

101. Wisting L, Siegwarth C, Skrivarhaug T, Dahl-Jørgensen K, Rø Ø. The impact of psychological aspects, age, and BMI on eating disorder psychopathology among adult males and females with type 1 diabetes. *Health Psychology Open* 2020; **7**(2): 2055102920975969.

102. Wolfgram P, Zhang L, Simpson P, Fiallo‐Scharer R. Clinical associations of quarterly Patient Health Questionnaire‐9 depression screening results in adolescents with type 1 diabetes. *Pediatric diabetes* 2020; **21**(5): 871-7.

103. Wu Y, Hilliard M, Rausch J, Dolan L, Hood K. Family involvement with the diabetes regimen in young people: the role of adolescent depressive symptoms. *Diabetic Medicine* 2013; **30**(5): 596-602.

104. Yayan EH, Zengin M, Akıncı A. The relationship between the quality of life and depression levels of young people with type I diabetes. *Perspectives in psychiatric care* 2019; **55**(2): 291-9.

105. Zaffani S, Maccagnan I, Morandi A, et al. Anxiety, Depression and Quality of Life in Italian Youths with Type 1 Diabetes Mellitus. *J Diabetes Metab* 2015; **6**(607): 2.

106. Zduńczyk B, Sendela J, Szypowska A. High prevalence of depressive symptoms in well‐controlled adolescents with type 1 diabetes treated with continuous subcutaneous insulin infusion. *Diabetes/metabolism research and reviews* 2014; **30**(4): 333-8.

107. Adams RN, Tanenbaum ML, Hanes SJ, et al. Psychosocial and human factors during a trial of a hybrid closed loop system for type 1 diabetes management. *Diabetes technology & therapeutics* 2018; **20**(10): 648-53.

108. Al Hayek AA, Robert AA, Al Dawish MA. Effectiveness of the freestyle libre flash glucose monitoring system on diabetes distress among individuals with type 1 diabetes: a prospective study. *Diabetes Therapy* 2020; **11**(4): 927-37.

109. Balfe M, Doyle F, Smith D, et al. What’s distressing about having type 1 diabetes? A qualitative study of young adults’ perspectives. *BMC Endocrine Disorders* 2013; **13**(1): 1-14.

110. Beverly EA, Rennie RG, Guseman EH, Rodgers A, Healy AM. High prevalence of diabetes distress in a university population. *The Journal of the American Osteopathic Association* 2019; **119**(9): 556-68.

111. Cechetti JV, Puñales M, da Cunha LZV, Rigo L. Emotional distress in patients with type 1 diabetes mellitus. *Spec Care Dentist* 2020; **40**(6): 589-96.

112. d'Emden H, McDermott B, D'Silva N, et al. Psychosocial screening and management of young people aged 18–25 years with diabetes. *Internal medicine journal* 2017; **47**(4): 415-23.

113. Danne T, Joish VN, Afonso M, et al. Improvement in Patient-Reported Outcomes in Adults with Type 1 Diabetes Treated with Sotagliflozin plus Insulin Versus Insulin Alone. *Diabetes technology & therapeutics* 2021; **23**(1): 70-7.

114. do Nascimento Andrade CJ, Alves CdAD. Influence of socioeconomic and psychological factors in glycemic control in young children with type 1 diabetes mellitus. *Jornal de pediatria* 2019; **95**(1): 48-53.

115. Ebrahimpour F, Sadeghi N, Najafi M, Iraj B, Shahrokhi A. Effect of playing interactive computer game on distress of insulin injection among type 1 diabetic children. *Iranian journal of pediatrics* 2015; **25**(3).

116. Evans MA, Weil LE, Shapiro JB, et al. Psychometric properties of the parent and child problem areas in diabetes measures. *Journal of pediatric psychology* 2019; **44**(6): 703-13.

117. Fegan‐Bohm K, Minard CG, Anderson BJ, et al. Diabetes distress and HbA1c in racially/ethnically and socioeconomically diverse youth with type 1 diabetes. *Pediatric Diabetes* 2020; **21**(7): 1362-9.

118. Fisher L, Polonsky WH, Hessler DM, et al. Understanding the sources of diabetes distress in adults with type 1 diabetes. *Journal of diabetes and its complications* 2015; **29**(4): 572-7.

119. Fisher L, Hessler D, Polonsky W, Strycker L, Masharani U, Peters A. Diabetes distress in adults with type 1 diabetes: prevalence, incidence and change over time. *Journal of diabetes and its complications* 2016; **30**(6): 1123-8.

120. Fisher L, Hessler DM, Polonsky W, et al. T1-REDEEM—An RCT to Reduce Diabetes Distress in Adults with Type 1 Diabetes. Am Diabetes Assoc; 2018.

121. Fisher L, Hessler D, Polonsky W, et al. Emotion regulation contributes to the development of diabetes distress among adults with type 1 diabetes. *Patient education and counseling* 2018; **101**(1): 124-31.

122. Griva F, Thomakos P, Kepaptsoglou O, et al. Internal structure and psychometric properties of Diabetes Distress Scale for Type 1 Diabetes. *Psychiatrike= Psychiatriki* 2020; **31**(4): 302-9.

123. Hansen UM, Skinner T, Olesen K, Willaing I. Diabetes distress, intentional hyperglycemia at work, and glycemic control among workers with type 1 diabetes. *Diabetes Care* 2019; **42**(5): 797-803.

124. Hansen UM, Olesen K, Willaing I. Diabetes stigma and its association with diabetes outcomes: a cross-sectional study of adults with type 1 diabetes. *Scand J Public Health* 2020; **48**(8): 855-61.

125. Al Hayek AA, Al Dawish MA. Assessing Diabetes Distress and Sleep Quality in Young Adults with Type 1 Diabetes Using FreeStyle Libre: A Prospective Cohort Study. *Diabetes Therapy* 2020; **11**: 1551-62.

126. Hessler D, Fisher L, Polonsky W, et al. Diabetes distress is linked with worsening diabetes management over time in adults with type 1 diabetes. *Diabetic Medicine* 2017; **34**(9): 1228-34.

127. Hessler D, Fisher L, Polonsky W, et al. There is value in treating elevated levels of diabetes distress: the clinical impact of targeted interventions in adults with Type 1 diabetes. *Diabetic medicine : a journal of the British Diabetic Association* 2020; **37**(1): 71-4.

128. Iturralde E, Weissberg-Benchell J, Hood KK. Avoidant coping and diabetes-related distress: pathways to adolescents’ type 1 diabetes outcomes. *Health Psychology* 2017; **36**(3): 236.

129. Iturralde E, Rausch JR, Weissberg-Benchell J, Hood KK. Diabetes-related emotional distress over time. *Pediatrics* 2019; **143**(6).

130. Joensen LE, Madsen KP, Holm L, et al. Diabetes and COVID-19: psychosocial consequences of the COVID-19 pandemic in people with diabetes in Denmark-what characterizes people with high levels of COVID-19-related worries? *Diabetic medicine : a journal of the British Diabetic Association* 2020; **37**(7): 1146-54.

131. Kelly CS, Berg CA, Helgeson VS. Adult attachment insecurity and associations with diabetes distress, daily stressful events and self-management in type 1 diabetes. *J Behav Med* 2020; **43**(5): 695-706.

132. Khan A, Choudhary P. Investigating the association between diabetes distress and self-management behaviors. *Journal of diabetes science and technology* 2018; **12**(6): 1116-24.

133. Knight BA, Hickman IJ, Gibbons K, Taylor J, McIntyre HD. Psychosocial outcomes in adults with type 1 diabetes following a novel ‘short course’structured flexible MDI therapy self‐management programme. *Practical Diabetes* 2017; **34**(7): 235-9a.

134. Kuniss N, Müller UA, Kloos C, Jörgens V, Kramer G. Diabetes-related distress is reduced in people with type 1, but not type 2 diabetes after participating in a diabetes treatment and teaching programme. *Diabetes & metabolism* 2019; **45**(3): 310-2.

135. Lašaitė L, Dobrovolskienė R, Danytė E, et al. Diabetes distress in males and females with type 1 diabetes in adolescence and emerging adulthood. *Journal of diabetes and its complications* 2016; **30**(8): 1500-5.

136. Lašaitė L, Ostrauskas R, Žalinkevičius R, Jurgevičienė N, Radzevičienė L. Diabetes distress in adult type 1 diabetes mellitus men and women with disease onset in childhood and in adulthood. *Journal of diabetes and its complications* 2016; **30**(1): 133-7.

137. Law GU, Walsh J, Queralt V, Nouwen A. Adolescent and parent diabetes distress in type 1 diabetes: The role of self-efficacy, perceived consequences, family responsibility and adolescent–parent discrepancies. *Journal of Psychosomatic Research* 2013; **74**(4): 334-9.

138. Li C, Ford ES, Zhao G, et al. Association between diagnosed diabetes and serious psychological distress among US adults: the Behavioral Risk Factor Surveillance System, 2007. *International journal of public health* 2009; **54**(1): 43-51.

139. Lee SL, Tsai MC, Chang SC, Chen JL, Wang RH. Modelling individual, parental and peer factors to glycaemic control in adolescents with type 1 diabetes: A prospective study. *Journal of advanced nursing* 2020; **76**(5): 1162-71.

140. Lohiya NN, Kajale NA, Lohiya NN, Khadilkar VV, Gondhalekar K, Khadilkar A. Diabetes distress in Indian children with type 1 diabetes mellitus and their mothers. *Journal of Pediatric Endocrinology and Metabolism* 2021; **34**(2): 209-16.

141. Luo J, Wang H, Li X, et al. Factors associated with diabetes distress among adolescents with type 1 diabetes. *Journal of clinical nursing* 2021.

142. Messer LH-S. Predictors of Continuous Glucose Monitoring Use in Adolescents with Type 1 Diabetes: University of Colorado Denver, Anschutz Medical Campus; 2019.

143. Miyawaki Y, Iwahashi H, Okauchi Y, et al. Differences in emotional distress among inpatients with type 1, obese type 2, and non-obese type 2 diabetes mellitus. *Internal Medicine* 2015; **54**(20): 2561-7.

144. Nouwen A, Urquhart Law G, Hussain S, McGovern S, Napier H. Comparison of the role of self-efficacy and illness representations in relation to dietary self-care and diabetes distress in adolescents with type 1 diabetes. *Psychology and health* 2009; **24**(9): 1071-84.

145. Oldham V, Mumford B, Lee D, Jones J, Das G. Impact of insulin pump therapy on key parameters of diabetes management and diabetes related emotional distress in the first 12 months. *Diabetes Res Clin Pract* 2020; **166**: 108281.

146. Polonsky WH, Anderson BJ, Lohrer PA, et al. Assessment of diabetes-related distress. *Diabetes care* 1995; **18**(6): 754-60.

147. Polonsky WH, Hessler D, Layne JE, Zisser H. Impact of the Omnipod® Insulin Management System on quality of life: a survey of current users. *Diabetes technology & therapeutics* 2016; **18**(10): 664-70.

148. Polonsky WH, Hessler D, Ruedy KJ, Beck RW. The impact of continuous glucose monitoring on markers of quality of life in adults with type 1 diabetes: further findings from the DIAMOND randomized clinical trial. *Diabetes Care* 2017; **40**(6): 736-41.

149. Polonsky WH, Fortmann AL. Impact of real-time continuous glucose monitoring data sharing on quality of life and health outcomes in adults with type 1 diabetes. *Diabetes technology & therapeutics* 2021; **23**(3): 195-202.

150. Powers MA, Richter SA, Ackard DM, Craft C. Diabetes distress among persons with type 1 diabetes: associations with disordered eating, depression, and other psychological health concerns. *The Diabetes Educator* 2017; **43**(1): 105-13.

151. Schmitt A, Reimer A, Kulzer B, Haak T, Ehrmann D, Hermanns N. How to assess diabetes distress: comparison of the Problem Areas in Diabetes Scale (PAID) and the Diabetes Distress Scale (DDS). *Diabetic Medicine* 2016; **33**(6): 835-43.

152. Snoek FJ, Pouwer F, Welch GW, Polonsky WH. Diabetes-related emotional distress in Dutch and US diabetic patients: cross-cultural validity of the problem areas in diabetes scale. *Diabetes care* 2000; **23**(9): 1305-9.

153. Snoek F, Van Der Ven N, Lubach C, et al. Effects of cognitive behavioural group training (CBGT) in adult patients with poorly controlled insulin-dependent (type 1) diabetes: a pilot study. *Patient education and counseling* 2001; **45**(2): 143-8.

154. Spaic T, Robinson T, Goldbloom E, et al. Closing the Gap: Results of the Multicenter Canadian Randomized Controlled Trial of Structured Transition in Young Adults With Type 1 Diabetes. *Diabetes Care* 2019; **42**(6): 1018-26.

155. Speight J, Holmes-Truscott E, Harvey DM, et al. Structured type 1 diabetes education delivered in routine care in Australia reduces diabetes-related emergencies and severe diabetes-related distress: The OzDAFNE program. *Diabetes research and clinical practice* 2016; **112**: 65-72.

156. Stahl-Pehe A, Glaubitz L, Bächle C, et al. Diabetes distress in young adults with early-onset Type 1 diabetes and its prospective relationship with HbA(1c) and health status. *Diabetic medicine : a journal of the British Diabetic Association* 2019; **36**(7): 836-46.

157. Stanulewicz N, Mansell P, Cooke D, Hopkins D, Speight J, Blake H. PAID-11: A brief measure of diabetes distress validated in adults with type 1 diabetes. *Diabetes research and clinical practice* 2019; **149**: 27-38.

158. Strandberg RB, Graue M, Wentzel‐Larsen T, Peyrot M, Thordarson HB, Rokne B. Longitudinal relationship between diabetes‐specific emotional distress and follow‐up HbA1c in adults with Type 1 diabetes mellitus. *Diabetic Medicine* 2015; **32**(10): 1304-10.

159. Todd PJ, Edwards F, Spratling L, et al. Evaluating the relationships of hypoglycaemia and HbA1c with screening‐detected diabetes distress in type 1 diabetes. *Endocrinology, diabetes & metabolism* 2018; **1**(1): e00003.

160. Van Beers CA, De Wit M, Kleijer SJ, et al. Continuous glucose monitoring in patients with type 1 diabetes and impaired awareness of hypoglycemia: also effective in patients with psychological distress? *Diabetes technology & therapeutics* 2017; **19**(10): 595-9.

161. Vesco AT, Jedraszko AM, Garza KP, Weissberg-Benchell J. Continuous glucose monitoring associated with less diabetes-specific emotional distress and lower A1c among adolescents with type 1 diabetes. *Journal of diabetes science and technology* 2018; **12**(4): 792-9.

162. Vesco AT, Feldman MA, Evans MA, Weissberg-Benchell J. Parent–adolescent dyadic diabetes distress: Associations with A1c and diabetes-related strengths. *Families, Systems, & Health* 2018; **36**(3): 357.

163. Weissberg‐Benchell J, Antisdel‐Lomaglio J. Diabetes‐specific emotional distress among adolescents: feasibility, reliability, and validity of the problem areas in diabetes‐teen version. *Pediatric diabetes* 2011; **12**(4pt1): 341-4.

164. Weissberg-Benchell J, Vesco AT, Rychlik K. Diabetes camp still matters: Relationships with diabetes-specific distress, strengths, and self-care skills. *Pediatr Diabetes* 2019; **20**(3): 353-60.

165. Wilmot EG, Close KL, Jurišić‐Eržen D, et al. Patient‐reported outcomes in adults with type 1 diabetes in global real‐world clinical practice: the SAGE study. *Diabetes, Obesity and Metabolism* 2021.

166. Abdoli S, Hessler D, Vora A, Smither B, Stuckey H. CE: Original Research: Experiences of Diabetes Burnout: A Qualitative Study Among People with Type 1 Diabetes. *AJN The American Journal of Nursing* 2019; **119**(12): 22-31.

167. Abdoli S, Jones DH, Vora A, Stuckey H. Improving diabetes care: should we reconceptualize diabetes burnout? *The Diabetes Educator* 2019; **45**(2): 214-24.

168. Abdoli S, Hessler D, Vora A, Smither B, Stuckey H. Descriptions of diabetes burnout from individuals with Type 1 diabetes: an analysis of YouTube videos. *Diabetic Medicine* 2020; **37**(8): 1344-51.

169. Helgeson VS. Diabetes burnout among emerging adults with type 1 diabetes: a mixed methods investigation. *Journal of Behavioral Medicine* 2021; **44**(3): 368-78.

170. Lowes L, Eddy D, Channon S, et al. The experience of living with type 1 diabetes and attending clinic from the perception of children, adolescents and carers: analysis of qualitative data from the DEPICTED study. *Journal of pediatric nursing* 2015; **30**(1): 54-62.

171. Blanchette JE, Toly VB, Wood JR. Financial stress in emerging adults with type 1 diabetes in the United States. *Pediatric Diabetes* 2021.

172. Due‐Christensen M, Zoffmann V, Hommel E, Lau M. Can sharing experiences in groups reduce the burden of living with diabetes, regardless of glycaemic control? *Diabetic Medicine* 2012; **29**(2): 251-6.

173. Fleer J, Tovote K, Keers J, et al. Screening for depression and diabetes‐related distress in a diabetes outpatient clinic. *Diabetic medicine* 2013; **30**(1): 88-94.

174. Hagger V, Hendrieckx C, Cameron F, Pouwer F, Skinner TC, Speight J. Diabetes distress is more strongly associated with HbA1c than depressive symptoms in adolescents with type 1 diabetes: Results from Diabetes MILES Youth—Australia. *Pediatric diabetes* 2018; **19**(4): 840-7.

175. Hapunda G, Abubakar A, Pouwer F, Van De Vijver F. Correlates of fear of hypoglycemia among patients with type 1 and 2 diabetes mellitus in outpatient hospitals in Zambia. *International Journal of Diabetes in Developing Countries* 2020; **40**(4): 619-26.

176. Holmes-Truscott E, Ventura AD, Thuraisingam S, Pouwer F, Speight J. Psychosocial Moderators of the Impact of Diabetes Stigma: Results From the Second Diabetes MILES - Australia (MILES-2) Study. *Diabetes Care* 2020; **43**(11): 2651-9.

177. Hood KK, Iturralde E, Rausch J, Weissberg-Benchell J. Preventing diabetes distress in adolescents with type 1 diabetes: results 1 year after participation in the STePS program. *Diabetes Care* 2018; **41**(8): 1623-30.

178. Hopkins D, Lawrence I, Mansell P, et al. Improved biomedical and psychological outcomes 1 year after structured education in flexible insulin therapy for people with type 1 diabetes: the UK DAFNE experience. *Diabetes care* 2012; **35**(8): 1638-42.

179. Kampling H, Mittag O, Herpertz S, Baumeister H, Kulzer B, Petrak F. Can trajectories of glycemic control be predicted by depression, anxiety, or diabetes-related distress in a prospective cohort of adults with newly diagnosed type 1 diabetes? Results of a five-year follow-up from the German multicenter diabetes cohort study (GMDC-Study). *Diabetes research and clinical practice* 2018; **141**: 106-17.

180. Liu J, Bispham J, Fan L, et al. Factors associated with fear of hypoglycaemia among the T1D Exchange Glu population in a cross-sectional online survey. *BMJ open* 2020; **10**(9): e038462.

181. McIntyre HD, Knight BA, Harvey DM, Noud MN, Hagger VL, Gilshenan KS. Dose adjustment for normal eating (DAFNE)—an audit of outcomes in Australia. *Medical Journal of Australia* 2010; **192**(11): 637-40.

182. Nguyen LA, Pouwer F, Winterdijk P, et al. Prevalence and course of mood and anxiety disorders, and correlates of symptom severity in adolescents with type 1 diabetes: Results from diabetes LEAP. *Pediatric Diabetes* 2021; **22**(4): 638-48.

183. Raymaekers K, Prikken S, Oris L, et al. A Person-Centered Perspective on the Role of Peer Support and Extreme Peer Orientation in Youth with Type 1 Diabetes: A Longitudinal Study. *Ann Behav Med* 2020; **54**(11): 893-903.

184. Raymaekers K, Helgeson VS, Prikken S, et al. Diabetes-specific friend support in emerging adults with type 1 diabetes: Does satisfaction with support matter? *Journal of Behavioral Medicine* 2021; **44**(3): 402-11.

185. Schmitt A, Reimer A, Kulzer B, Haak T, Gahr A, Hermanns N. Negative association between depression and diabetes control only when accompanied by diabetes-specific distress. *Journal of behavioral medicine* 2015; **38**(3): 556-64.

186. Schmitt A, Bendig E, Baumeister H, Hermanns N, Kulzer B. Associations of depression and diabetes distress with self-management behavior and glycemic control. *Health Psychology* 2020.

187. Silveira M, Neto AM, Sposito A, Siminerio L, Pavin E. Low empowerment and diabetes regimen distress are related to HbA1c in low income type 1 diabetes patients in a Brazilian tertiary public hospital. *Diabetology & metabolic syndrome* 2019; **11**(1): 1-8.

188. Snoek F, Van Der Ven N, Twisk J, et al. Cognitive behavioural therapy (CBT) compared with blood glucose awareness training (BGAT) in poorly controlled Type 1 diabetic patients: long‐term effects on HbA1c moderated by depression. A randomized controlled trial. *Diabetic Medicine* 2008; **25**(11): 1337-42.

189. Strandberg RB, Graue M, Wentzel-Larsen T, Peyrot M, Rokne B. Relationships of diabetes-specific emotional distress, depression, anxiety, and overall well-being with HbA1c in adult persons with type 1 diabetes. *Journal of psychosomatic research* 2014; **77**(3): 174-9.

190. Tanenbaum ML, Gonzalez JS. The influence of diabetes on a clinician-rated assessment of depression in adults with type 1 diabetes. *The Diabetes Educator* 2012; **38**(5): 695-704.

191. Weissberg-Benchell J, Rausch J, Iturralde E, Jedraszko A, Hood K. A randomized clinical trial aimed at preventing poor psychosocial and glycemic outcomes in teens with type 1 diabetes (T1D). *Contemporary clinical trials* 2016; **49**: 78-84.

192. Weissberg-Benchell J, Shapiro JB, Bryant FB, Hood KK. Supporting Teen Problem-Solving (STEPS) 3 year outcomes: Preventing diabetes-specific emotional distress and depressive symptoms in adolescents with type 1 diabetes. *J Consult Clin Psychol* 2020; **88**(11): 1019-31.

193. Wong J, Addala A, Naranjo D, et al. Monetary reinforcement for self‐monitoring of blood glucose among young people with type 1 diabetes: evaluating effects on psychosocial functioning. *Diabetic Medicine* 2020; **37**(4): 665-73.

194. Wong JJ, Hanes S, Iturralde E, et al. Do Youth Want Psychosocial Screenings in Diabetes Clinic? Profiles of Acceptability. *Journal of pediatric psychology* 2021; **46**(3): 332-40.

195. Younes ZM, Abuali AM, Tabba S, Farooqi MH, Hassoun AA. Prevalence of diabetes distress and depression and their association with glycemic control in adolescents with type 1 diabetes in Dubai, United Arab Emirates. *Pediatric diabetes* 2021; **22**(4): 683-91.

196. Zajdel M, Helgeson VS, Kelly CS, Berg CA. Shared illness appraisal and self-efficacy among adults with type 1 diabetes. *J Health Psychol* 2021; **26**(3): 390-400.

197. Zhu L, Chandran SR, Tan WB, Xin X, Goh S-Y, Gardner DS-L. Persistent anxiety is associated with higher glycemia post-transition to adult services in Asian Young adults with diabetes. *Diabetes & metabolism journal* 2020; **45**(1): 67-76.

198. Abdoli S, Miller-Bains K, Burr EM, Smither B, Vora A, Hessler D. Burnout, distress, and depressive symptoms in adults with type 1 diabetes. *Journal of diabetes and its complications* 2020; **34**(7): 107608.

199. Abdoli S, Hessler D, Smither B, Miller-Bains K, Burr EM, Stuckey HL. New insights into diabetes burnout and its distinction from diabetes distress and depressive symptoms: A qualitative study. *Diabetes Research and Clinical Practice* 2020; **169**: 108446.

200. Abdoli S, Miller-Bains K, Fanti P, Silveira M, Hessler D. Development and validation of a scale to measure diabetes burnout. *J Clin Transl Endocrinol* 2021; **23**: 100251.

201. Abdoli S, Silveira MS, Doosti-Irani M, et al. Cross-national comparison of psychosocial well-being and diabetes outcomes in adults with type 1 diabetes during the COVID-19 pandemic in US, Brazil, and Iran. *Diabetology & metabolic syndrome* 2021; **13**(1): 1-12.
