# Supplementary material for: Unraveling the concepts of distress, burnout, and depression in type 1 diabetes: A scoping review
Source: eClinicalMedicine. 2021 Aug 28;40:101118. doi: 10.1016/j.eclinm.2021.101118 (PMC8408521; doi:10.1016/j.eclinm.2021.101118)
Supplement: Supplementary file 2 [file mmc2.docx]

**Supplementary Figure 1. Number of publications by regions of the world**

Bar length represents the number of publications by region

Supplementary Figure 2. **Circular bar plot of concepts of depression, diabetes distress and diabetes burnout by authors’ descriptions**

Bar length represents the frequency of words or phrases for each concept in percentage among the 201 included studies. Words or phrases found in studies about depression, diabetes distress and diabetes burnout are in blue, green and salmon respectively. Abbreviations: Detach.from sup.system: Detachment from support system

Supplementary Figure 3. Circular bar plot of concepts of depression, diabetes distress and diabetes burnout by questionnaire items

Bar length represents the frequency of words or phrases for each concept in percentage among the 201 included studies. Words or phrases found in studies about depression, diabetes distress and diabetes burnout are in blue, green and salmon respectively. Abbreviations: Detach.from sup.system: Detachment from support system; Detach. from.illness id.: Detachment from illness identification; Uncomfort.social: Uncomfortable in social settings

**Supplementary Figure 4. Word Clouds for depression, diabetes distress and diabetes burnout.**

Word size reflects how often the word was cited among the studies included by each concept. Words with an equivalent citation frequency share the same colour.
